# Resuscitation fluid use in critically ill adults: an international cross-sectional study in 391 intensive care units

**DOI:** 10.1186/cc9293

**Published:** 2010-10-15

**Authors:** Simon Finfer, Bette Liu, Colman Taylor, Rinaldo Bellomo, Laurent Billot, Deborah Cook, Bin Du, Colin McArthur, John Myburgh

**Affiliations:** 1 Critical Care and Trauma Division, The George Institute for International Health, PO Box M201, Missenden Road, NSW 2050, Australia; 2 Faculty of Medicine, University of New South Wales, NSW 2052, Australia; 3 Department of Intensive Care, Austin Hospital, 145 Studley Rd, Heidelberg, Melbourne, VIC 3084, Australia; 4 Departments of Medicine, Clinical Epidemiology & Biostatistics, McMaster University, 1200 Main St West, Hamilton, ON L8N 3Z5, Canada; 5 Director of Medical ICU, Peking Union Medical College Hospital, Peking Union Medical College, 1 Shuai Fu Yuan, Beijing 100730, China; 6 Department of Critical Care Medicine, Auckland City Hospital, Park Road, Grafton, Auckland 1023, New Zealand

## Abstract

**Introduction:**

Recent evidence suggests that choice of fluid used for resuscitation may influence mortality in critically ill patients.

**Methods:**

We conducted a cross-sectional study in 391 intensive care units across 25 countries to describe the types of fluids administered during resuscitation episodes. We used generalized estimating equations to examine the association between patient, prescriber and geographic factors and the type of fluid administered (classified as crystalloid, colloid or blood products).

**Results:**

During the 24-hour study period, 1,955 of 5,274 (37.1%) patients received resuscitation fluid during 4,488 resuscitation episodes. The main indications for administering crystalloid or colloid were impaired perfusion (1,526/3,419 (44.6%) of episodes), or to correct abnormal vital signs (1,189/3,419 (34.8%)). Overall, colloid was administered to more patients (1,234 (23.4%) versus 782 (14.8%)) and during more episodes (2,173 (48.4%) versus 1,468 (32.7%)) than crystalloid. After adjusting for patient and prescriber characteristics, practice varied significantly between countries with country being a strong independent determinant of the type of fluid prescribed. Compared to Canada where crystalloid, colloid and blood products were administered in 35.5%, 40.6% and 28.3% of resuscitation episodes respectively, odds ratios for the prescription of crystalloid in China, Great Britain and New Zealand were 0.46 (95% confidence interval (CI) 0.30 to 0.69), 0.18 (0.10 to 0.32) and 3.43 (1.71 to 6.84) respectively; odds ratios for the prescription of colloid in China, Great Britain and New Zealand were 1.72 (1.20 to 2.47), 4.72 (2.99 to 7.44) and 0.39 (0.21 to 0.74) respectively. In contrast, choice of fluid was not influenced by measures of illness severity (for example, Acute Physiology and Chronic Health Evaluation (APACHE) II score).

**Conclusions:**

Administration of resuscitation fluid is a common intervention in intensive care units and choice of fluid varies markedly between countries. Although colloid solutions are more expensive and may possibly be harmful in some patients, they were administered to more patients and during more resuscitation episodes than crystalloids were.

## Introduction

Administration of intravenous fluid is one of the most common interventions in the management of patients in intensive care units (ICUs). Despite this, there is limited high quality information to guide clinicians in deciding when fluid resuscitation may be indicated and what type of fluid to prescribe [1-3]. Reports from clinicians suggest that the type of fluid used for resuscitation varies widely [4-6] but there is little evidence regarding what fluids are administered and the factors that influence the type of fluid prescribed. We conducted an international cross-sectional study of intensive care units to examine these issues.

## Materials and methods

### Design and setting

A total of 391 intensive care units (ICUs) from 25 countries contributed data to the study (hereafter referred to as contributing ICUs). The contributing ICUs were recruited through convenience sampling using the personal contacts of the investigators and by contacting the leaders of critical care research networks. All institutions that obtained ethical approval according to the local requirements were included. For the purposes of this observational study, as there was no deviation from routine medical care, ethical board approval was either waved or expedited at all sites and individual patient informed consent was not required. Data were collected for all patients present in the contributing ICUs for all or part of a single 24-hour study day in 2007. Depending on local logistic considerations each contributing ICU chose the 18 or 25 April, 16 May, 20 June or 11 July as their study day.

### Participants and data collection

Data were collected on a standard data collection form (see Additional file 1) and this was returned to the study co-ordinating centre for entry into the study database. Data were checked against pre-specified range limits and any queries were resolved with the study sites. For each patient, information was collected on their sex, age, ICU admission date, admission source and diagnosis (based on the Acute Physiology and Chronic Health Evaluation (APACHE) II [7] diagnosis codes) and whether resuscitation fluid or blood products were prescribed. An episode of fluid resuscitation was defined as an hour during which either a bolus of crystalloid or colloid; a crystalloid infusion of 5 ml/kg/hr or greater; a continuous infusion of colloid at any dose; or any volume of whole blood, packed red blood cells, fresh frozen plasma or platelets was given.

For patients who received at least one episode of fluid resuscitation during the study period additional information was collected from the patient's medical record. This included the patient's weight, APACHE II score [7], the presence of trauma as the primary ICU admission diagnosis, and based on standard definitions, the presence of traumatic brain injury [8], severe sepsis [9] or acute respiratory distress syndrome [10]. For each episode of fluid resuscitation the types of fluids and volumes infused, cardiovascular and respiratory components of the Sequential Organ Failure Assessment (SOFA) score [11], clinical signs (heart rate, mean arterial pressure, central venous pressure), most recent laboratory measures (haemoglobin, creatinine, bilirubin, lactate, and albumin concentrations), urine output and total fluid output in the previous complete hour, use of renal replacement therapy and mechanical ventilation were recorded. The type of fluid given for resuscitation was classified into crystalloid, colloid (with colloid sub-classification as albumin, starch, gelatin or dextran solutions), or blood products (whole blood, packed cells, platelets or fresh frozen plasma) as indicated on the standard data collection form (Additional file 1). The indication for the fluid, the specialty and seniority of the fluid prescriber, and the type of fluid prescribed was recorded by the bedside nurse at the time of the resuscitation episode. For episodes where more than one indication for fluid resuscitation was provided, these were classified according to a predetermined hierarchy (see Additional File 2). Data on mortality or discharge at 28 days following ICU admission were also collected to characterise the study population.

### Statistical analysis

Patients aged less than 16 years were excluded from analyses. As more than one type of fluid could have been administered during one fluid resuscitation episode, where proportions of episodes are given they may add to more than 100%; also separate analyses were conducted for each type of fluid, that is, crystalloid given or not, colloid given or not, blood product given or not. The association between a patient's demographic and clinical characteristics and the type of fluid administered were analysed using generalized estimating equations. Initially each factor of interest was examined separately to determine if there was an association with the type of fluid administered. Factors found to have a predetermined level of association (*P *< 0.1) with the administration of crystalloid, colloid or blood product were then included in multivariate analyses. In the multivariate analysis, a conservative approach was taken and associations were considered significant if *P *< 0.01.

In analyses, for categorical data with no natural order, the reference group was selected based on the category with the greatest number of observations except for country, where Canada was selected because the pattern of fluid use in Canada most closely resembled that of all the contributing ICUs combined. Countries (or territories) with less than 100 episodes of resuscitation were combined into two categories, 'other European countries' (Iceland, Republic of Ireland and Northern Ireland, Norway and Portugal) and 'other countries' (Brazil, India, Japan, Saudi Arabia, Singapore and United Arab Emirates). Analyses were conducted using STATA 9.2 statistical software (Stata Corp LP, College Station, Texas, USA).

## Results

**Figure 1:**
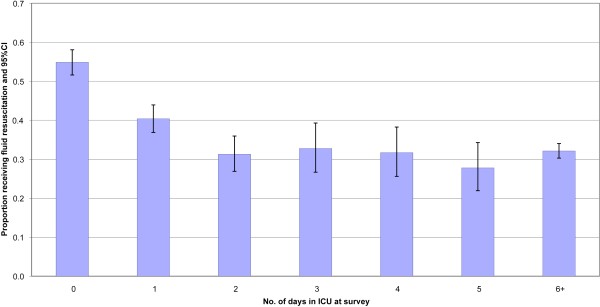
**Proportion of study participants receiving fluid resuscitation according to the number of days in the ICU**.

After excluding 62 patients aged less than 16 years or of unknown age, a total of 5,274 patients were included. Table 1 shows the contributing countries and within each of these the number of contributing ICUs, patients and fluid resuscitation episodes. Overall 37.1% (1,955) of patients received fluid resuscitation during the 24-hour study period. This percentage was higher in patients for whom the study period coincided with their admission date to ICU; specifically 55% and 40% of patients who were surveyed on respectively Day 0 or Day 1 in the ICU received fluid resuscitation (Figure 1). Of the patients who received fluid, 848 (43.4%) received fluid in one hour only, 495 (25.3%) in two separate hours and 612 (31.3%) in three separate hours or more. Among those receiving fluid, the median number of hours where fluid was administered was two (mean two, inter-quartile range one to three).

**Table 1:** Countries/territories, intensive care units and patients included in the survey

	No of ICUs	No patients surveyed	No patients given fluid resuscitation	% patients given fluid resuscitation	No episodes of fluid resuscitation
Australia	24	402	142	35.3	346
Brazil	5	92	14	15.2	20
Canada	20	420	163	38.8	434
China	57	1,129	503	44.6	962
Denmark	17	133	65	48.9	146
France	42	583	110	18.9	214
Germany	23	535	253	47.3	675
Great Britain*	38	390	145	37.2	350
Hong Kong	6	95	46	48.4	109
Iceland	3	14	11	78.6	24
India	1	31	8	25.8	17
Ireland and N. Ireland	8	94	29	30.9	70
Italy	71	514	158	30.7	297
Japan	5	47	17	36.2	28
New Zealand	9	104	36	34.6	148
Norway	19	112	46	41.1	88
Portugal	1	13	1	7.7	3
Saudi Arabia	4	78	26	33.3	57
Singapore	3	35	13	37.1	27
Sweden	24	177	71	40.1	189
Switzerland	6	77	38	49.4	118
United Arab Emirates	1	17	6	35.3	16
USA	4	182	54	29.7	150
**Total**	**391**	**5,274**	**1,955**	**37.1**	**4,488**

*includes England, Scotland and Wales

**Figure 2:**
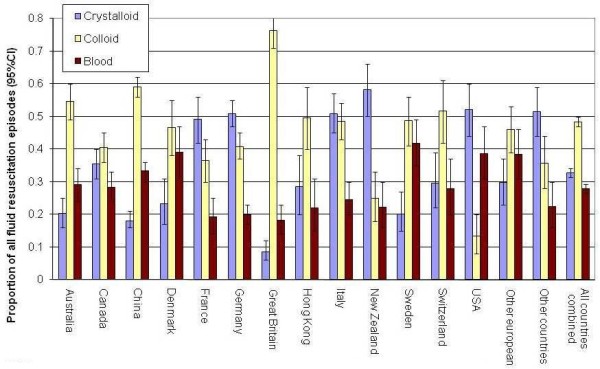
Percentage of fluid resuscitation episodes given as crystalloid, colloid or blood product according to country*. Crystalloid; Colloid; Blood: *Difference in proportions given crystalloid, colloid or blood between countries, respectively *P *< 0.001, *P *< 0.001, *P *< 0.001

**Figure 3:**
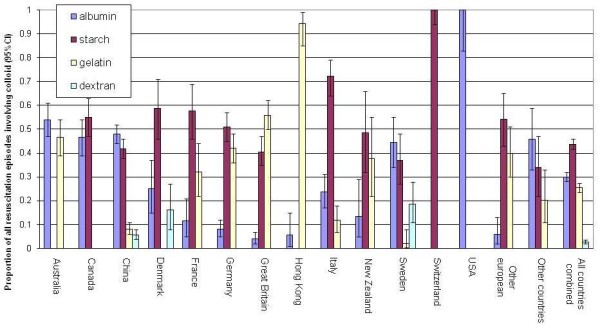
**Type of colloid used as a percentage of all colloid episodes by country**. Albumin; starch; gelatin; dextran

Figures 2 and 3 show by country the proportion of fluid resuscitation episodes given as crystalloid, colloid and blood product, and the types of colloid as a proportion of all episodes where colloid was given, respectively. Overall crystalloid was administered during 33% of resuscitation episodes, colloid during 48% of episodes and blood products during 28%. Between countries the percentage of episodes where crystalloid was administered ranged from 9 to 58%, colloid from 13 to 76% and blood products from 18 to 42%. The type of colloid used for fluid resuscitation in the contributing ICUs also differed between countries (Figure 3); overall starch was administered in 44% of colloid resuscitation episodes, albumin in 30%, gelatin in 25% and dextran in 3%.

The characteristics of the 1,955 patients who received fluid resuscitation during the study are shown in Table 2. Of the 4,488 episodes of fluid resuscitation, 39.2% were for the indication of impaired perfusion or low cardiac output. The majority of other fluid resuscitation episodes were for abnormal vital signs in the absence of impaired perfusion (28.5%) or for anaemia, bleeding or coagulopathy (18.5%). Considering only episodes where crystalloid or colloid were administered (*n *= 3,419), the main indications were impaired perfusion or low cardiac output (44.6%), or to correct abnormal vital signs in the absence of impaired perfusion or low cardiac output (34.8%).

**Table 2:** Characteristics of 1,955 patients who received fluid resuscitation and indications for the 4,488 episodes of fluid resuscitation

Demographic characteristics	*N *= 1955	
Age, yrs (mean, SD)	60.8 (17.4)	
Sex (% male, N)	64.3 (1,256)	
		
**Admission characteristics**		
No of days in ICU at survey date (median, IQR)	3 (0, 10)	
Hospital admission for trauma (%, N)	12.0 (234)	
Sepsis in 24 hrs prior to survey date (%, N)	33.1 (642)	
APACHE II in 24 hrs prior to survey date (mean, SD)	16.3 (8.5)	
Died in ICU/hospital at ≤ 28 days (%, N)	16.1 (304)	
Admission source		
% Operating room after elective surgery	25.1 (489)	
% Hospital floor	22.2 (432)	
% Transfer from other ICU or hospital	11.8 (230)	
% Operating room after emergency surgery	14.5 (283)	
% Emergency room	17.0 (331)	
% Hospital floor after previous ICU stay	9.4 (184)	
		
**Indication for fluid in each fluid resuscitation episode**	*N *= 4,488	
% Impaired perfusion/low cardiac output (n)	39.2 (1,743)	
% Abnormal vital signs	28.5 (1,266)	
% Anaemia/bleeding/coagulopathy	18.5 (822)	
% Unit protocol	6.6 (294)	
% Other fluid losses	2.9 (131)	
% Other	4.3 (191)	

Percentages are calculated as column percents. Numbers do not necessarily add to totals due to missing values.

The patient characteristics, clinical signs and prescriber factors associated with administration of crystalloid, colloid or blood products, are shown by patient and by episode of fluid resuscitation in Tables 3 and 4 respectively. After adjusting for factors that were found by univariate analysis to be associated (*P *< 0.1) with the administration of crystalloid, colloid or blood product, significant associations remained (Table 5). The type of fluid prescribed in the contributing ICUs differed significantly between countries. Compared to Canada (where the proportion of all fluid episodes prescribed as crystalloid, colloid and blood products was 35.5%, 40.6% and 28.3% respectively, and the distribution most closely resembled that of all contributing ICUs combined) crystalloid was less likely to be administered to patients in contributing ICUs in China, Great Britain and Sweden, but more likely to be administered to patients in contributing ICUs in Italy, New Zealand and the USA; there was no significant difference in crystalloid prescription to patients in contributing ICUs between Canada and Australia, Denmark, France, Germany, Hong Kong and Switzerland. Conversely, compared to Canada, colloid was more likely to be administered to patients in contributing ICUs in China and Great Britain and less likely to be administered to patients in contributing ICUs in New Zealand and the USA. Blood was significantly more likely to be prescribed in contributing ICUs in China, Denmark, Sweden and the USA compared to contributing ICUs in Canada.

**Table 3:** Characteristics of 1,955 study participants who received crystalloid, colloid or blood products (patients who recevied more than one fluid type are included more than once)

	Crystalloid			Colloid			Blood Products		
	Given (*N *= 782)	Not given (*N *= 1,173)	*P*-value*	Given (*N *= 1,234)	Not given (*N *= 721)	*P*-value	Given (*N *= 717)	Not given (*N *= 1,238)	*P*-value
**Patient characteristic**									
Age, yrs (mean, SD)	60.3 (17.7)	61.2 (17.1)	0.3	61.7 (16.9)	59.3 (18.1)	0.003	60.0 (17.9)	61.3 (17.1)	0.09
Sex (% male, n)	64.3 (502)	64.3 (754)	0.99	64.6 (797)	63.7 (459)	0.7	64.2 (460)	64.4 (796)	0.9
Number of days in ICU at survey date (median, IQR)	1 (0,8)	4 (1,11)	< 0.001	3 (0,10)	3 (0,11)	0.6	3 (1,10)	2 (0,10)	0.09
APACHE II score in 24 hrs prior to survey date (mean, SD)	16.5 (8.7)	16.1 (8.4)	0.3	16.2 (8.6)	16.4 (8.4)	0.7	17.0 (8.7)	15.9 (8.4)	0.004
									
**Patient characteristic: comparison of percentage of patients given or not given each fluid type who had the following characteristic**									
Hospital admission for trauma, *n *= 234% (N)	14.4 (112)	10.4 (122)	0.009	11.0 (135)	13.8 (99)	0.07	13.7 (98)	11.0 (136)	0.08
Hospital admission for trauma with brain injury, *n *= 81% (N)	5.2 (41)	3.4 (40)	0.05	3.8 (47)	4.7 (34)	0.3	4.9 (35)	3.7 (46)	0.2
Sepsis in 24 hrs prior to survey date, *n *= 642% (N)	27.8 (216)	36.6 (426)	< 0.001	36.4 (445)	27.5 (197)	< 0.001	33.3 (237)	33.0 (405)	0.9
APACHE II in 24 hrs prior to survey date > 15, *n *= 957% (N)	49.6 (384)	49.7 (573)	0.96	48.4 (588)	51.9 (369)	0.1	54.2 (381)	47.1 (576)	0.003
APACHE II Chronic health points									
Chronic health points liver criteria, *n *= 107% (N)	5.2 (40)	5.8 (67)	0.6	6.1 (74)	4.6 (33)	0.2	6.1 (43)	5.3 (64)	0.4
Chronic health points renal criteria, *n *= 88% (N)	4.4 (34)	4.7 (54)	0.8	4.0 (49)	5.5 (39)	0.1	5.5 (39)	4.0 (49)	0.1
Chronic health points cardiac criteria, *n *= 151% (N)	7.8 (60)	7.9 (91)	0.96	8.4 (102)	6.9 (49)	0.2	7.9 (56)	7.8 (95)	0.9
Chronic health points respiratory criteria, *n *= 203% (N)	10.8 (83)	10.4 (120)	0.8	10.6 (128)	10.5 (75)	0.97	9.6 (68)	11.1 (135)	0.3
Chronic health points immunocompromised, *n *= 164% (N)	6.9 (53)	9.6 (111)	0.04	8.2 (100)	9.0 (64)	0.6	10.3 (73)	7.5 (91)	0.03
									
**Admission source: comparison of percentage of patients from each source given or not given each fluid type**									
Admission source			< 0.001			< 0.001			0.02
Operating room after elective surgery, *n *= 489% (N)	38.5 (188)	61.6 (301)		67.3 (329)	32.7 (160)		40.1 (196)	59.9 (293)	
Hospital floor, *n *= 432% (N)	31.9 (138)	68.1 (294)		67.1 (290)	32.9 (142)		37.0 (160)	63.0 (272)	
Transfer from other ICU or hospital, *n *= 230% (N)	38.3 (88)	61.7 (142)		57.8 (133)	42.2 (97)		38.7 (89)	61.3 (141)	
Operating room after emergency surgery, *n *= 283% (N)	43.8 (124)	56.2 (159)		67.8 (192)	32.2 (91)		35.3 (100)	64.7 (183)	
Emergency room, *n *= 331% (N)	55.0 (182)	45.0 (149)		53.8 (178)	46.2 (153)		28.7 (95)	71.3 (236)	
Hospital floor after previous ICU stay, *n *= 184% (N)	31.5 (58)	68.5 (126)		58.2 (107)	41.9 (77)		40.8 (75)	59.2 (109)	

*P-*values highlighted where < 0.1 and hence eligible to be included in multivariate analysis

*P-values calculated using chi2 for proportions, t-tests for means and Kruskal Wallis test for non-parametric data

**Table 4:** Comparison of indication for fluid, seniority of fluid prescriber and patient characteristics present in relation to administration of crystalloid, colloid or blood by episode for 4,488 fluid resuscitation episodes in 1,955 study participants

		Crystalloid			Colloid			Blood		
		Given *N *= 1,468	Not given *N *= 3,020	*P*-value	Given *N *= 2,173	Not given *N *= 2,315	*P*-value	Given *N *= 1,249	Not given *N *= 3,239	*P*-value
Indication for fluid										
Impaired perfusion or low cardiac output, *n *= 1,743% (N)		48.0 (739)	34.3 (1,004)	< 0.001	41.6 (899)	35.6 (844)	< 0.001	27.0 (330)	42.7 (1,413)	< 0.001
Anaemia/bleeding/coagulopathy, *n *= 822% (N)		4.9 (68)	23.1 (754)		5.5 (119)	29.0 (703)		49.5 (717)	3.9 (105)	
Other fluid losses, *n *= 131% (N)		3.4 (49)	2.8 (82)		2.9 (64)	3.1 (67)		2.3 (23)	3.2 (108)	
Unit protocol, *n *= 294% (N)		5.9 (74)	8.2 (220)		9.9 (201)	4.9 (93)		3.8 (36)	8.7 (258)	
Abnormal vital signs, *n *= 1,266% (N)		33.5 (483)	26.0 (783)		34.9 (751)	21.9 (515)		8.5 (100)	35.5 (1,166)	
Other, *n *= 191% (N)		3.1 (44)	5.3 (147)		5.8 (117)	3.3 (74)		2.9 (32)	5.2 (159)	
Fluid prescriber										
ICU Specialist/consultant, *n *= 1,495% (N)		35.0 (482)	36.3 (1,013)	< 0.001	35.2 (690)	36.6 (805)	0.2	40.3 (486)	34.3 (1,009)	< 0.001
ICU Registrar, *n *= 1,368% (N)		27.1 (353)	31.7 (1,015)		30.9 (710)	29.6 (658)		30.2 (395)	30.3 (973)	
ICU Resident/junior staff, *n *= 1,063% (N)		21.4 (431)	20.5 (632)		21.4 (497)	20.2 (566)		19.9 (235)	21.1 (828)	
Other, *n *= 562% (N)		14.6 (202)	11.9 (360)		12.5 (276)	13.0 (286)		10.8 (133)	13.5 (429)	
Cardiac failure	SOFA < 3, *n *= 2,973% (N)			0.1			0.08			0.2
	SOFA 3+, *n *= 1,443% (N)	25.6 (459)	27.9 (984)		27.6 (706)	26.6 (737)		28.0 (429)	26.8 (1014)	
Respiratory failure	SOFA < 3, *n *= 2,793% (N)			0.01			0.02			0.1
	SOFA 3+, *n *= 1,471% (N)	31.4 (420)	34.6 (1,051)		34.7 (774)	32.4 (697)		34.8 (436)	33.2 (1035)	
Renal replacement therapy	No, *n *= 4,048% (N)			0.02			0.3			< 0.001
	Yes, *n *= 417% (N)	8.7 (103)	10.2 (314)		9.5 (233)	10.1 (184)		10.9 (175)	9.3 (242)	
Mechanical ventilation	No, *n *= 1,568% (N)			0.04			0.1			0.02
	Yes, *n *= 2,907% (N)	60.5 (910)	62.8 (1,997)		62.9 (1,438)	61.2 (1,469)		63.5 (845)	61.5 (2,062)	
Low filling pressure**	No, *n *= 1,448% (N)			0.001			0.8			0.1
	Yes, *n *= 1,277% (N)	52.7 (430)	45.7 (847)		47.6 (652)	47.7 (625)		45.9 (331)	48.3 (946)	
Metabolic acidosis**	No, *n *= 3,062% (N)			0.02			0.7			0.9
	Yes, *n *= 800% (N)	18.8 (279)	17.3 (521)		17.4 (372)	18.0 (428)		17.5 (243)	17.8 (557)	
Lactate (mmol/L)**	< 2, *n *= 1,851% (N)			0.3			0.6			0.07
	2+, *n *= 1,222% (N)	37.0 (441)	36.5 (781)		36.9 (554)	36.5 (668)		37.2 (391)	36.5 (831)	
Heart rate (mean, b/min)		95	94	0.07	94	94	0.8	93	94	0.9
Mean arterial pressure (mean, mmHg)		76	77	0.3	75	78	< 0.001	79	76	< 0.001
Haemoglobin (mean, g/L)		100	97	< 0.001	100	96	< 0.001	92	100	< 0.001
Creatinine (mean, umol/L)		148	142	0.06	141	147	0.002	146	143	0.2
Bilirubin** (mean, umol/L)		38	39	0.96	39	38	0.7	45	40	0.1
Albumin** (mean, g/L)		27	27	0.9	27	27	0.3	27	27	0.9
Urine output** (mean, ml/kg/hr)		2.0	1.8	0.1	1.8	1.8	0.99	2.0	1.8	0.02
Fluid output** (mean, ml/kg/hr)		2.9	2.8	0.9	2.8	2.8	0.6	3.1	2.7	0.008

*P*-values highlighted where < 0.1 and hence eligible to be included in multivariate analysis.

Proportions and means adjusted for repeated measures in individuals with more than one episode of fluid resuscitation. Proportions do not include missing values. Numbers do not necessarily add to totals due to missing values.

**Variables have > 10% missing values

**Table 5:** Multivariate analysis of factors associated with the use of crystalloid, colloid or blood for fluid resuscitation episodes*

	OR (95%CI) crystalloid given		OR (95%CI) colloid given		OR (95%CI) blood given	
Characteristic		*P*-value		*P*-value		*P*-value
Age (per one year increase)	1.00 (0.99 to 1.00)		1.00 (1.00 to 1.01)		1.00 (0.99 to 1.01)	
Study region						
Canada	1.00		1.00		1.00	
Australia	0.69 (0.42 to 1.11)		1.33 (0.88 to 2.02)		1.79 (1.00 to 3.23)	
China	0.46 (0.30 to 0.69)	< 0.001	1.72 (1.20 to 2.47)	0.003	3.33 (2.02 to 5.50)	< 0.001
Denmark	0.50 (0.27 to 0.94)		1.43 (0.83 to 2.48)		4.09 (2.03 to 8.25)	< 0.001
France	1.60 (0.96 to 2.66)		0.82 (0.51 to 1.32)		0.88 (0.43 to 1.78)	
Germany	1.62 (1.08 to 2.44)		0.79 (0.54 to 1.14)		1.07 (0.62 to 1.84)	
Great Britain	0.18 (0.10 to 0.32)	< 0.001	4.72 (2.99 to 7.44)	< 0.001	0.93 (0.51 to 1.73)	
Hong Kong	0.88 (0.44 to 1.74)		0.93 (0.51 to 1.72)		1.17 (0.47 to 2.93)	
Italy	2.06 (1.28 to 3.31)	0.003	1.33 (0.86 to 2.06)		1.10 (0.59 to 2.03)	
New Zealand	3.43 (1.71 to 6.84)	< 0.001	0.39 (0.21 to 0.74)	0.004	0.48 (0.19 to 1.24)	
Sweden	0.43 (0.23 to 0.80)	0.008	1.67 (0.99 to 2.82)		4.99 (2.58 to 9.63)	< 0.001
Switzerland	0.96 (0.48 to 1.92)		1.12 (0.60 to 2.10)		1.18 (0.49 to 2.82)	
USA	2.55 (1.36 to 4.79)	0.004	0.16 (0.08 to 0.33)	< 0.001	3.81 (1.78 to 8.17)	0.001
Other European	1.01 (0.59 to 1.72)		1.26 (0.77 to 2.05)		2.27 (1.17 to 4.39)	
Other countries	2.52 (1.46 to 4.33)	0.001	0.53 (0.32 to 0.89)		1.43 (0.70 to 2.93)	
Admission source						
Operating room after elective surgery	1.00		1.00		1.00	
Hospital floor	1.26 (0.91 to 1.75)		1.05 (0.80 to 1.38)		0.59 (0.41 to 0.84)	0.003
Transfer from other ICU or hospital	1.49 (1.03 to 2.15)		0.80 (0.58 to 1.11)		0.60 (0.39 to 0.91)	
Operating room after emergency surgery	1.57 (1.12 to 2.20)	0.008	0.93 (0.69 to 1.24)		0.58 (0.39 to 0.86)	0.006
Emergency room	2.16 (1.56 to 2.99)	< 0.001	0.67 (0.50 to 0.90)	0.008	0.50 (0.34 to 0.75)	0.001
Hospital floor after previous ICU stay	1.12 (0.75 to 1.67)		1.18 (0.83 to 1.67)		0.62 (0.39 to 0.99)	
Trauma at hospital admission						
No trauma	1.00		1.00		1.00	
Trauma without brain injury	0.93 (0.64 to 1.35)		1.02 (0.73 to 1.42)		1.26 (0.82 to 1.95)	
Trauma with brain injury	1.13 (0.69 to 1.84)		1.05 (0.68 to 1.64)		1.15 (0.63 to 2.08)	
Sepsis in 24 hrs prior to survey date						
No sepsis	1.00		1.00		1.00	
Sepsis	0.90 (0.71 to 1.15)		1.26 (1.02 to 1.55)		0.67 (0.51 to 0.89)	0.005
Number of days in ICU at survey date						
0 days	1.00		1.00		1.00	
> 0 days	0.70 (0.56 to 0.87)	0.001	1.28 (1.05 to 1.56)		0.91 (0.70 to 1.19)	
Indication for fluid						
Impaired perfusion or low cardiac output	1.00		1.00		1.00	
Anaemia/bleeding/coagulopathy	0.15 (0.12 to 0.20)	< 0.001	0.13 (0.10 to 0.17)	< 0.001	26.7 (20.2 to 35.4)	< 0.001
Other fluid losses	0.90 (0.60 to 1.37)		0.83 (0.55 to 1.23)		0.90 (0.54 to 1.49)	
Unit protocol	0.72 (0.52 to 1.01)		1.65 (1.21 to 2.25)	0.002	0.44 (0.29 to 0.67)	< 0.001
Abnormal vital signs	0.93 (0.78 to 1.11)		1.34 (1.12 to 1.60)	0.001	0.34 (0.25 to 0.44)	< 0.001
Other	0.39 (0.26 to 0.59)	< 0.001	1.70 (1.18 to 2.45)	0.004	0.74 (0.47 to 1.18)	
Fluid prescriber						
ICU Specialist/consultant	1.00		1.00		1.00	
ICU Registrar	0.88 (0.71 to 1.09)		1.10 (0.90 to 1.34)		0.83 (0.64 to 1.08)	
ICU Resident/junior staff	1.19 (0.95 to 1.49)		1.04 (0.83 to 1.29)		0.72 (0.53 to 0.96)	
Other	1.36 (1.05 to 1.76)		1.12 (0.87 to 1.45)		0.60 (0.42 to 0.85)	0.004
Cardiovascular dysfunction						
SOFA < 3	1.00		1.00		1.00	
SOFA 3 to 4	0.94 (0.77 to 1.14)		1.08 (0.89 to 1.29)		1.26 (0.99 to 1.61)	
Respiratory failure						
SOFA < 3	1.00		1.00		1.00	
SOFA 3 to 4	0.87 (0.72 to 1.06)		1.10 (0.92 to 1.33)		1.02 (0.80 to 1.31)	
Renal replacement therapy						
No	1.00		1.00		1.00	
Yes	0.81 (0.59 to 1.11)		0.81 (0.60 to 1.08)		1.86 (1.29 to 2.68)	0.001
Mechanical ventilation						
No	1.00		1.00		1.00	
Yes	0.91 (0.75 to 1.12)		1.02 (0.85 to 1.23)		1.32 (1.02 to 1.71)	
Low filling pressure						
No	1.00		1.00		1.00	
Yes	1.26 (1.02 to 1.55)		0.86 (0.70 to 1.05)		1.08 (0.83 to 1.40)	
Heart rate (per 10 b/min increase)	1.05 (1.01 to 1.09)		0.98 (0.95 to 1.02)		1.01 (0.96 to 1.06)	
Mean arterial pressure	0.97 (0.93 to 1.02)		1.16 (1.11 to 1.21)	< 0.001	0.85 (0.80 to 0.91)	< 0.001
(per 10 mmHg decrease)						
Haemoglobin (per 10 g/L decrease)	0.97 (0.93 to 1.01)		0.94 (0.91 to 0.98)	0.004	1.24 (1.18 to 1.30)	< 0.001
% 100+	1.00		1.00		1.00	
% 80 to 99	0.91 (0.75 to 1.10)		0.92 (0.77 to 1.10)		1.80 (1.39 to 2.31)	< 0.001
% 70 to 79	0.72 (0.54 to 0.98)		0.89 (0.68 to 1.18)		3.24 (2.29 to 4.57)	< 0.001
% < 70	0.75 (0.52 to 1.09)		0.60 (0.43 to 0.84)	0.004	5.74 (3.84 to 8.58)	< 0.001
Creatinine						
(per 10 umol/L increase)	1.00 (1.00 to 1.01)		1.00 (0.99 to 1.00)		1.00 (1.00 to 1.01)	
< 170 umol/L	1.00		1.00		1.00	
170+umol/L	0.86 (0.66 to 1.11)		1.00 (0.79 to 1.26)		1.22 (0.91 to 1.63)	
Chronic health points immunocompromised						
No	1.00		1.00		1.00	
Yes	0.64 (0.44 to 0.93)		1.32 (0.96 to 1.83)		0.96 (0.64 to 1.45)	
APACHE II 24 hrs prior to fluid administration						
per 1 point increase in score	1.01 (1.00 to 1.03)		0.99 (0.98 to 1.01)		1.01 (0.99 to 1.02)	
< 16	1.00		1.00		1.00	
16+	1.13 (0.91 to 1.41)		0.88 (0.72 to 1.07)		1.30 (1.00 to 1.68)	
Lactate** (mmol/L)						
< 2	1.00		1.00		1.00	
2+	1.02 (0.82 to 1.26)		0.90 (0.74 to 1.11)		1.59 (1.21 to 2.08)	0.001
Urine output** (ml/kg/hr)						
per ml/kg/hr	1.03 (1.00 to 1.06)		1.03 (0.99 to 1.06)		0.98 (0.93 to 1.02)	
< 0.5	1.00		1.00		1.00	
0.5+	0.89 (0.72 to 1.09)		1.10 (0.89 to 1.35)		1.03 (0.77 to 1.38)	
Total fluid output** (ml/kg/hr)						
per ml/kg/hr	1.01 (0.99 to 1.02)		1.01 (0.99 to 1.03)		1.01 (0.98 to 1.03)	
< 1	1.00		1.00		1.00	
1+	1.17 (0.95 to 1.43)		1.03 (0.85 to 1.24)		1.02 (0.79 to 1.33)	

*P*-values highlighted where < 0.01

Analyses include 4,430 episodes and 1939 study participants as data were lost due to missing values which could not be included in the multivariate analysis due to small numbers in cells. This number represents a loss of 1.5% of episodes and 1.1% of study participants.

**Variables have > 10% missing values

Other than country of location of the contributing ICUs, few factors were independently associated with the administration of crystalloid. Elective post-operative patients were more likely to receive colloid than crystalloid (67.3% versus 38.5%). Compared to this group, those admitted after emergency surgery or from the emergency department were more likely to be resuscitated with crystalloid (OR = 1.57, 95% CI 1.12 to 2.20 and OR = 2.16, 95% CI 1.56 to 2.99 respectively). Among the 514 patients who were admitted to the ICU on the study day, colloid was also more commonly prescribed than crystalloid (622/1,395 episodes (44.6%) versus 561/1,395 (40.2%)). Compared to this group, those who had been in the ICU for longer were less likely to receive crystalloid (OR = 0.70, 95% CI 0.56 to 0.87). In patients where the indication for fluid was impaired perfusion or low cardiac output, colloid was administered more commonly than crystalloid (899/1,743 (51.6%) versus 739/1,743 (42.4%)) and compared to this group, colloid was more likely to be administered as part of a unit protocol (OR 1.65, 95% CI 1.21 to 2.25) and for correction of abnormal vital signs (OR 1.34, 95% CI 1.12 to 1.60). For episodes where the indication was anaemia, bleeding or coagulopathy, administration of crystalloid or colloid was less likely (OR = 0.15, 95% CI 0.12 to 0.20 and OR = 0.13, 95% CI 0.10 to 0.17 respectively) and blood products more likely (OR = 26.7, 95% CI 20.2 to 35.4). The likelihood of receiving colloid increased significantly with a lower mean arterial pressure (OR = 1.16, 95% CI 1.11 to 1.21 per 10 mmHg decrease).

The administration of blood products was predominantly determined by two factors, a reported indication of 'anaemia, bleeding or coagulopathy' (OR 26.7, 95% CI 20.2 to 35.4 compared to 'impaired perfusion or low cardiac output') and haemoglobin concentration (OR 1.24, 95% CI 1.18 to 1.30 per 10 g/L decrease). Patients being treated with renal replacement therapy (OR 1.86, 95% CI 1.29 to 2.68) and with hyperlactaemia (OR 1.59, 95% CI 1.21 to 2.08) were also more likely to receive blood products. Blood products were less likely to be prescribed if the patient had severe sepsis diagnosed in the 24 hours prior to the survey (OR 0.67, 95% CI 0.51 to 0.89). Compared to patients admitted to the ICU following elective surgery, blood was less likely to be prescribed in those admitted from the hospital floor, the operating theatre after emergency surgery or the emergency room.

## Discussion

In this large international study of fluid resuscitation in ICUs we found that administration of resuscitation fluids was very common. For patients who were surveyed on their first day in the ICU, over half received resuscitation fluid and overall more than a third of all ICU patients received resuscitation fluid on the study day. The main indications given for fluid resuscitation were "impaired perfusion" or to "correct abnormal vital signs". Overall colloids were administered to more patients and during more resuscitation episodes than were crystalloids; the country in which the patient was being treated was a major determinant of fluid choice even after adjusting for patient and prescriber characteristics.

This was a large pragmatic survey of the actual fluid administered in intensive care. It covered a number of ICUs from different countries and used standard data collection forms and definitions. Detailed information on many of the factors that may influence the choice of fluid for resuscitation were recorded at the time that fluid was given, allowing analyses to take into account many potentially important patient and prescriber characteristics. Our conclusions are limited by the fact that we used a convenience sample, and the findings may not be universally applicable. For instance, some regions, such as Europe or Australasia, contributed greater numbers of patients to the study, and hence where findings are reported for the entire population, they may more accurately reflect practice in these regions. In addition, we could not account for all possible factors that may influence fluid prescription such as fluid that was prescribed before the study day, nor could we adjust our analyses for the individual practitioners or institutions.

A strength of our study is that we collected data on fluids actually administered and related these to geographic, patient and prescriber characteristics. This methodology is more reliable than previous studies that have asked practitioners which fluids they prefer without documenting actual use. Practitioner surveys conducted in European countries [4,6] and Canada [5] suggest that the fluid used for resuscitation varies substantially across different countries and there is little consistency with respect to preferred fluids for particular patient groups or clinical scenarios. Our results show that the actual fluids administered vary as much as clinicians' stated preferences.

This study was conducted in 2007 and we found that in most countries colloids were used more commonly than crystalloids. Preceding the study there was limited evidence regarding appropriate indications for fluid resuscitation [12,13]. The largest randomised controlled trial had compared resuscitation with albumin or saline in 6,997 critically ill patients; it reported no substantial difference in any important patient-centred outcome [14]. The Cochrane meta-analyses [2] current at the time of our survey concluded that use of colloids was hard to justify outside the context of a randomised controlled trial. More recent evidence suggests that human albumin increases mortality in patients with traumatic brain injury [8] and that some hydroxyethyl starch solutions may increase the incidence of renal failure in patients with severe sepsis [15,16]. Observational data suggest that this risk might extend to use of other colloids [17].

In the face of emerging evidence that choice of resuscitation fluid may affect important patient outcomes, particularly in subgroups of critically ill patients, our findings have important implications for clinicians, researchers and policy makers. They suggest that many clinicians are guided predominantly by local practice and in many regions colloids are used widely and preferentially for indications such as "unit protocol" or "abnormal vital signs in the absence of impaired perfusion"; this has the potential to increase costs and to harm some patients. Our results also imply that current research evidence may not be considered robust enough to override local custom and practice; those researching in the field of fluid resuscitation should concentrate on conducting randomised controlled trials of sufficient size and methodological rigour to change clinical practice. Policy makers should be aware that practice varies widely by country and is guided more by local practice than by reliable research evidence. Given that pharmaceutical agents have to demonstrate safety and efficacy before they receive marketing approval, regulatory agencies should consider reviewing their criteria for granting marketing approval to resuscitation fluids. In particular they should require evidence from clinical trials that examine longer term patient-centred safety and efficacy outcomes.

It is possible that for many patients the choice of resuscitation fluid does not significantly affect outcome. If this is true, then the practice variation apparent in our survey may be of academic and economic interest only. However, as resuscitation fluids are administered to so many critically ill adults each day, relatively small differences in benefit, harm or cost per patient will result in large effects overall. Further large-scale clinical trials are needed both to examine the effects of particular fluids, and also to determine the appropriate indications for the administration of resuscitation fluids.

## Conclusions

Fluid resuscitation is a common intervention in critically ill patients. Recent evidence suggests that the use of colloids may be harmful in some subgroups of critically ill patients but there are few reliable data about what fluids patients receive and what factors influence fluid choice. This study shows that the choice of fluid varies substantially between ICUs and geographic location appears to be a strong determinant of practice variation that is not explained by patient factors. Despite evidence of superiority being lacking and increased cost, in this survey colloids were more frequently administered to resuscitate critically ill patients than crystalloids.

## Key messages

• Close to 40% of patients in intensive care units receive resuscitation fluids each day.

• In this large international survey colloid resuscitation fluids were used more often than crystalloids.

• The fluid used varies substantially between ICUs and local practice rather than patient characteristics appears to be the main factor in fluid choice.

## Abbreviations

APACHE: Acute Physiology and Chronic Health Evaluation; SOFA: Sequential Organ Failure Assessment

## Competing interests

CSL Ltd. partially funded the original SAFE Study and has refunded travel expenses incurred by SF and RB in presenting the results at industry sponsored and academic meetings. Fresenius Kabi has refunded travel expenses incurred by SF and JM in attending meetings to discuss research into the clinical effects of hydroxyethyl starches in critically ill patients. Fresenius Kabi has provided an unrestricted research grant to the University of Sydney for the conduct of a fluid resuscitation trial for which JM is the chief investigator. BL owns shares in CSL Ltd. BD has received speaker fees from B. Braun Medical (Shanghai) Co., Ltd, and Beijing Frensius Kabi Pharmaceutical Co., Ltd. CT, LB, CM and DC have no competing interests.

## Authors' contributions

SF conceived the study, supervised the design and conduct, and helped to draft the manuscript. BL contributed to the data analysis plan, conducted analyses and drafted the manuscript. CT co-ordinated and managed the study, contributed to the data analysis plan and helped to draft the manuscript. RB, DC, CM and JM contributed to study design and supervised the conduct of the study. LB advised on and supervised the data analysis plan. All authors critically reviewed the manuscript for important intellectual content and approved the final manuscript.

## Supplementary Material

Additional file 1 **Data collection forms: Case report form and other data collection forms used in The SAFE TRIPS Study**. Click here for file

Additional file 2 **Hierarchy for indications: Hierarchy of indications for administration of resuscitation fluid. Were more than one indication was given the indication highest on the hierarchy was taken to be the main indication**. Click here for file

Additional file 3 **The SAFE TRIPS Investigators: Listing of all SAFE TRIPS investigators by country and institution**. Click here for file
