# Supplementary material for: Resuscitation fluid use in critically ill adults: an international cross-sectional study in 391 intensive care units
Source: Crit Care. 2010 Oct 15;14(5):R185. doi: 10.1186/cc9293 (PMC3219291; doi:10.1186/cc9293)
Supplement: Additional file 1 — Data collection forms: Case report form and other data collection forms used in The SAFE TRIPS Study. [file cc9293-S1.DOCX]

**Additional File 1**: Data collection forms

| **SAFE TRIPS** | **ICU ADMISSION DATA – ALL PATIENTS FORM 1** |
| --- | --- |

| ***GENERAL PATIENT INFORMATION*** |
| --- |

| **1.01** | M | F | | | | Patient’s sex | | | | |
| --- | --- | --- | --- | --- | --- | --- | --- | --- | --- | --- |
| **1.02** | I__I__I__I | | | | | | Patient’s age (years) | | | |
| **1.03** | I__I__I/I__I__I/I__I__I__I__I | | | | | | | | ICU admission date (dd/mm/yyyy) | |
| **1.04** | I__I__I:I__I__I | | | | | | | ICU admission time (use 24 hour clock) | | |
| **1.05** | From where was the patient admitted to the ICU? | | | | | | | | | |
|  | Y | | | Accident and Emergency Department | | | | | | |
|  | Y | | | Hospital Floor | | | | | | |
|  | Y | | | Transfer from another ICU | | | | | | tick one box only |
|  | Y | | | Transfer from another hospital (except from another ICU) | | | | | |  |
|  | Y | | | Admitted from Operating Theatre following **EMERGENCY** surgery | | | | | | |
|  | Y | | | Admitted from Operating Theatre following **ELECTIVE** surgery | | | | | | |
| **1.06** | Y | | N | | Has this patient previously been in ICU in **THIS** hospital during **THIS** hospital admission? | | | | | |

| **yes** | | | | | | **no** | |  | | | | |
| --- | --- | --- | --- | --- | --- | --- | --- | --- | --- | --- | --- | --- |
| **1.07** | | | Y | | | N | Was this a **POST-OPERATIVE** admission to ICU? (Answer **yes** if patient admitted **DIRECT** from the operating theatre or the recovery room) | | | | | |
|  | | |  | | | | | If **no**, go to question **1.09** | | | | |
| **1.08** | | |  | | | | | If **yes**, what was the *primary* **POST-OPERATIVE** diagnosis that necessitated this admission to ICU? (tick one box only) | | | | |
|  |  | | | | | | **Cardiovascular:** | | | |  |  |
|  |  | | | | | | Y | Dissecting/ruptured aorta | | | |  |
|  |  | | | | | | Y | Peripheral vascular disease - no bypass graft | | | |  |
|  |  | | | | | | Y | Peripheral artery bypass graft | | | |  |
|  |  | | | | | | Y | Elective abdominal aortic aneurysm | | | |  |
|  |  | | | | | | Y | Carotid endarterectomy | | | |  |
|  |  | | | | | | Y | Valvular heart surgery | | | |  |
|  |  | | | | | | Y | Other cardiovascular disease | | | |  |
|  |  | | | | | | **Gastrointestinal:** | | | |  |  |
|  |  | | | | | | Y | Perforation / rupture | | | |  |
|  |  | | | | | | Y | Inflammatory disease | | | |  |
|  |  | | | | | | Y | Bleeding | | | |  |
|  |  | | | | | | Y | Obstruction | | | |  |
|  |  | | | | | | Y | Neoplasm | | | |  |
|  |  | | | | | | Y | Cholecystitis / cholangitis | | | |  |
|  |  | | | | | | Y | Liver transplant | | | |  |
|  |  | | | | | | Y | Other gastrointestinal diseases | | | |  |
|  |  | | | | | | **Respiratory:** | | | |  |  |
|  |  | | | | | | Y | Respiratory infection | | | |  |
|  |  | | | | | | Y | Neoplasm of lung | | | |  |
|  |  | | | | | | Y | Neoplasm – mouth / larynx / sinus / trachea | | | |  |
|  |  | | | | | | Y | Other respiratory diseases | | | |  |
|  |  | | | | | | **Neurological:** | | |  | |  |
|  |  | | | | | | Y | Intracerebral haemorrhage | | | |  |
|  |  | | | | | | Y | Subdural / epidural haematoma | | | |  |
|  |  | | | | | | Y | Subarachnoid haemorrhage | | | |  |
|  |  | | | | | | Y | Laminectomy / spinal cord injury | | | |  |
|  |  | | | | | | Y | Craniotomy for neoplasm | | | |  |
|  |  | | | | | | Y | Other neurologic disease | | | |  |
|  |  | | | | | | **Trauma:** | | | |  |  |
|  |  | | | | | | Y | Head trauma + / - multiple trauma | | | |  |
|  |  | | | | | | Y | Multiple trauma excluding head | | | |  |
|  |  | | | | | | Y | Burns | | | |  |
|  |  | | | | | | Y | Multiple trauma + spinal cord injury | | | |  |
|  |  | | | | | | **Renal:** | | | |  |  |
|  |  | | | | | | Y | Renal neoplasm | | | |  |
|  |  | | | | | | Y | Other renal diseases | | | |  |
|  |  | | | | | | **Gynaecological:** | | | |  |  |
|  |  | | | | | | Y | Hysterectomy | | | |  |
|  |  | | | | | | Y | Pregnancy related disorder | | | |  |
|  |  | | | | | | **Orthopaedic:** | |  | | |  |
|  |  | | | | | | Y | Hip or extremity disorder | | | |  |
| **1.09** | | | | What was the *primary* **MEDICAL** diagnosis that necessitated this admission to ICU? (tick one box only) | | | | | | | | |
|  | | | | |  | | | **Cardiovascular:** | | | | |
|  | | | | |  | | | Y | Cardiogenic shock | | | |
|  | | | | |  | | | Y | Cardiac arrest | | | |
|  | | | | |  | | | Y | Aortic aneurysm | | | |
|  | | | | |  | | | Y | Congestive cardiac failure | | | |
|  | | | | |  | | | Y | Peripheral vascular disease - medical | | | |
|  | | | | |  | | | Y | Rhythm disturbance | | | |
|  | | | | |  | | | Y | Acute myocardial infarction | | | |
|  | | | | |  | | | Y | Hypertension | | | |
|  | | | | |  | | | Y | Other non-surgical cardiovascular disease | | | |
|  | | | | |  | | | **Sepsis:** | | | | |
|  | | | | |  | | | Y | Sepsis other than urinary tract | | | |
|  | | | | |  | | | Y | Sepsis of urinary tract origin | | | |
|  | | | | |  | | | **Trauma:** | | | | |
|  | | | | |  | | | Y | Head trauma with or without multiple trauma | | | |
|  | | | | |  | | | Y | Multiple trauma excluding head | | | |
|  | | | |  | | | **Respiratory:** | | | |  |  |
|  | | | |  | | | Y | Aspiration pneumonia | | | |  |
|  | | | |  | | | Y | Respiratory neoplasm including larynx / trachea | | | |  |
|  | | | |  | | | Y | Respiratory arrest | | | |  |
|  | | | |  | | | Y | Pulmonary oedema (non-cardiac) | | | |  |
|  | | | |  | | | Y | Bacterial / viral pneumonia | | | |  |
|  | | | |  | | | Y | Chronic obstructive pulmonary disease | | | |  |
|  | | | |  | | | Y | Pulmonary embolism | | | |  |
|  | | | |  | | | Y | Mechanical airway obstruction | | | |  |
|  | | | |  | | | Y | Asthma | | | |  |
|  | | | |  | | | Y | Parasitic pneumonia | | | |  |
|  | | | |  | | | Y | Other non-surgical respiratory diseases | | | |  |
|  | | | |  | | | **Gastrointestinal:** | | |  | |  |
|  | | | |  | | | Y | Hepatic failure | | | |  |
|  | | | |  | | | Y | Perforation / obstruction | | | |  |
|  | | | |  | | | Y | Bleeding – varices | | | |  |
|  | | | |  | | | Y | Inflammatory disease (ulcerative colitis, Crohn’s, pancreatitis) | | | |  |
|  | | | |  | | | Y | Bleeding – ulceration / laceration | | | |  |
|  | | | |  | | | Y | Bleeding – diverticulitis | | | |  |
|  | | | |  | | | Y | Other non-surgical gastro-intestinal disease | | | |  |
|  | | | |  | | | **Neurological:** | | | |  |  |
|  | | | |  | | | Y | Intracerebral haemorrhage | | | |  |
|  | | | |  | | | Y | Subarachnoid haemorrhage | | | |  |
|  | | | |  | | | Y | Stroke | | | |  |
|  | | | |  | | | Y | Neurologic infection | | | |  |
|  | | | |  | | | Y | Neurologic neoplasm | | | |  |
|  | | | |  | | | Y | Neuromuscular disease | | | |  |
|  | | | |  | | | Y | Seizure | | | |  |
|  | | | |  | | | Y | Other neurological disease | | | |  |
|  | | | |  | | | **Metabolic:** | | | |  |  |
|  | | | |  | | | Y | Metabolic coma | | | |  |
|  | | | |  | | | Y | Diabetic ketoacidosis | | | |  |
|  | | | |  | | | Y | Drug overdose | | | |  |
|  | | | |  | | | Y | Other metabolic disease | | | |  |
|  | | | |  | | | **Haematological:** | | | |  |  |
|  | | | |  | | | Y | Coagulopathy / Neutro / Thrombo | | | |  |
|  | | | |  | | | Y | Other haematological diseases | | | |  |
|  | | | |  | | | **Renal:** | | | | |  |
|  | | | |  | | | Y | Renal disease | | | |  |
|  | | | |  | | | **Other:** | | | | |  |
|  | | | |  | | | Y | Other medical diseases | | | |  |

***If the patient received fluid for resuscitation on the study day, complete forms 2-5***

***Otherwise, the data collection is complete for this patient***

| **SAFE TRIPS** | **ICU ADMISSION DATA – ALL PATIENTS FORM 2** |
| --- | --- |

| ***GENERAL PATIENT INFORMATION*** |
| --- |

| **2.01** | I__I__I__I | | | | | Patient’s weight (in kg) | | |
| --- | --- | --- | --- | --- | --- | --- | --- | --- |
| **2.02** | Was the above weight estimated or measured? | | | | | | | |
|  | Y | | | | Estimated | | | |
|  | Y | | | | Measured | | | |
| **TRAUMA** | | | | | | | |  |
| **2.03** | | Y | N | | | Was the patient’s primary reason for hospital admission trauma (include burns or any type of trauma including falls in the elderly)? |  |  |
|  | |  | | | | If **no**, go to question **2.11** | |  |
|  | |  | | | | If **yes**, go to question **2.04** | |  |
|  | | Which of the following criteria for TRAUMA did the patient meet? (refer to manual for definitions) | | | | | |  |
| **2.04** | | Y | | N | | - An injury to the body produced by mechanical forces | |  |
| **2.05** | | Y | | N | | - A primary admission diagnosis of burns | |  |
|  | |  | |  | | If **no**, go to question **2.07** | |  |
|  | |  | | | | If **yes**, answer question **2.06** | |  |
| **2.06** | | I__I__I__I | | | | What was the percentage of body area of burns? | |  |
| **2.07** | | I__I__I | | | | What was the last GCS prior to sedation? | |  |
| **2.08** | | Was the GCS recorded in the patient record or estimated from a description of the patient’s neurological state? | | | | | |  |
|  | | Y | | | Recorded | | |  |
|  | | Y | | | Estimated | | |  |
| **2.09** | | Y | N | | | Was a cranial CT scan performed prior to ICU admission? | |  |
|  | |  |  | | | If **no**, go to question **2.11** | |  |
|  | |  |  | | | If **yes**, answer question **2.10** | |  |
| **2.10** | | Y | N | | | Was there an abnormality on cranial CT consistent with acute traumatic brain injury? | |  |

Complete the remaining questions for form 2 using the information from the 24 hours prior to the first resuscitation episode.

| **SEPSIS AT BASELINE** | | | | |
| --- | --- | --- | --- | --- |
| **2.11** | Y | N | Did patient meet BOTH of the following criteria for sepsis (refer to manual for definitions) | |
|  | | | | - a defined focus of infection (positive cultures not required) |
|  | | | | - 2 or more of the **S**ystemic **I**nflammatory **R**esponse **S**yndrome criteria |
|  | | | | - - Core temperature >38°C or <36°C. |
|  | | | | - - WCC >12 x 10^9^/L or < 4 x 10^9^/L or > 10% immature neutrophils (Band forms) |
|  | | | | - - Tachycardia - Heart rate >90 beats/minute |
|  | | | | - - Tachypnoea - >20 breaths per minute or a PaCO_2_ <32 mmHg or mechanical ventilation |

| **ACUTE RESPIRATORY DISTRESS SYNDROME (ARDS) AT BASELINE** | | | |
| --- | --- | --- | --- |
| **2.12** | Y | N | Did patient meet ALL of the following criteria for ARDS. (refer to manual for definitions) |
|  |  |  | - Bilateral diffuse alveolar infiltrates |
|  |  |  | - A recognised risk factors for ARDS |
|  |  |  | - Wedge pressure < 18mmHg or NO left atrial hypertension on clinical grounds |
|  |  |  | - PaO_2_/FiO_2_ < 200 |

| **APACHE II score** | | | | | |
| --- | --- | --- | --- | --- | --- |
| **2.13** | I__I__I | What was the APACHE II score for the 24 hours prior to the first resuscitation episode? (use an APACHE II worksheet to derive the score) | | | |
| **2.14** | I__I | What was the chronic health points score (part C); If the patient had chronic health points, indicate all that apply below: | | | |
|  |  | Y | N | Liver | Biopsy proven cirrhosis & documented portal hypertension (PH); episodes of upper GI bleeding due to PH; or prior episodes of hepatic failure/encephalopathy/coma |
|  |  | Y | N | Renal | Receiving chronic dialysis |
|  |  | Y | N | Cardiovascular | New York Heart Association Class IV – symptoms at rest |
|  |  | Y | N | Respiratory | Chronic restrictive, obstructive or vascular disease resulting in severe exercise restriction (i.e. unable to climb stairs, perform household duties); or documented chronic hypoxia, hypercapnia, 2^o^ polycythemia, severe pulmonary hypertension (>40mmHg) or respiratory dependency |
|  |  | Y | N | Immunocompromised | Patient has received therapy that suppresses resistance to infection, eg. immuno-suppression, chemotherapy, radiotherapy, long term or recent high dose steroids, or has a disease sufficiently advanced to suppress resistance to infection (eg leukaemia, lymphoma, AIDS) |

| **SAFE TRIPS** | **FLUID RESUSCITATION FORM 3** |
| --- | --- |

| ***RESUSCITATION EPISODE*** | | | | | | ***Episode 1*** | | | | | ***Episode 2*** | | | | | | | ***Episode 3*** | | | | | | |
| --- | --- | --- | --- | --- | --- | --- | --- | --- | --- | --- | --- | --- | --- | --- | --- | --- | --- | --- | --- | --- | --- | --- | --- | --- |
| **3.01** | | Start time of resuscitation episode(24 hour clock) | | | | I__I__I:I__I__I | | | | | I__I__I:I__I__I | | | | | | | I__I__I:I__I__I | | | | | | |
| What were the indications for fluid for this resuscitation episode? (tick boxes to define) | | | | | | | | | | | | | | | | | | | | | | | | |
| **3.02** | | Hypotension | | | | Y | | | N | | | Y | | | | N | | | Y | | | | N | |
| **3.03** | | Increasing inotrope or vasopressor requirements | | | | Y | | | N | | | Y | | | | N | | | Y | | | | N | |
| **3.04** | | Low CVP | | | | Y | | | N | | | Y | | | | N | | | Y | | | | N | |
| **3.05** | | Low PCWP | | | | Y | | | N | | | Y | | | | N | | | Y | | | | N | |
| **3.06** | | Tachycardia | | | | Y | | | N | | | Y | | | | N | | | Y | | | | N | |
| **3.07** | | Low urine output | | | | Y | | | N | | | Y | | | | N | | | Y | | | | N | |
| **3.08** | | Low measured cardiac output | | | | Y | | | N | | | Y | | | | N | | | Y | | | | N | |
| **3.09** | | Low Hb concentration | | | | Y | | | N | | | Y | | | | N | | | Y | | | | N | |
| **3.10** | | Low S_v_O_2_/S_cv_O_2_ | | | | Y | | | N | | | Y | | | | N | | | Y | | | | N | |
| **3.11** | | Ongoing bleeding | | | | Y | | | N | | | Y | | | | N | | | Y | | | | N | |
| **3.12** | | Other ongoing fluid loss | | | | Y | | | N | | | Y | | | | N | | | Y | | | | N | |
| **3.13** | | Unit protocol or standing orders | | | | Y | | | N | | | Y | | | | N | | | Y | | | | N | |
| **3.14** | | Evidence of poor peripheral perfusion | | | | Y | | | N | | | Y | | | | N | | | Y | | | | N | |
| **3.15** | | Increasing or persisting acidosis or lactate | | | | Y | | | N | | | Y | | | | N | | | Y | | | | N | |
| **3.16** | | Other, Specify _____________________ | | | | Y | | | N | | | Y | | | | N | | | Y | | | | N | |
| **3.17** | | Who decided the choice of fluid for this resuscitation episode? (tick one box only per resuscitation episode) | | | | | | | | | | | | | | | | | | | | | | |
|  | | ICU doctor | | | | Y | | | | | Y | | | | | | | Y | | | | | | |
|  | | Surgical doctor | | | | Y | | | | | Y | | | | | | | Y | | | | | | |
|  | | Medical doctor | | | | Y | | | | | Y | | | | | | | Y | | | | | | |
|  | | Nurse acting independently | | | | Y | | | | | Y | | | | | | | Y | | | | | | |
|  | | Nurse following unit protocol | | | | Y | | | | | Y | | | | | | | Y | | | | | | |
|  | | Other | | | | Y | | | | | Y | | | | | | | Y | | | | | | |
| **3.18** | | If you chose ICU, surgical or medical doctor on **3.17**, specify the doctor’s level (tick one box only per resuscitation episode); Otherwise, go to question **3.19** | | | | | | | | | | | | | | | | | | | | | | |
|  | | Specialist/Consultant/Attending | | | | Y | | | | | Y | | | | | | | Y | | | | | | |
|  | | Registrar/Fellow/Senior Trainee | | | | Y | | | | | Y | | | | | | | Y | | | | | | |
|  | | Resident/HMO/Junior Trainee | | | | Y | | | | | Y | | | | | | | Y | | | | | | |
|  | | Intern/House officer | | | | Y | | | | | Y | | | | | | | Y | | | | | | |
| ***RESUSCITATION EPISODE*** | | | | ***Episode 1*** | | | | | ***Episode 2*** | | | | | | | ***Episode 3*** | | | | | | | |  |
| **3.19** | | SOFA score – **respiration** | | I__I | | | | | I__I | | | | | | | I__I | | | | | | | |  |
| **3.20** | | SOFA score – **cardiovascular** | | I__I | | | | | I__I | | | | | | | I__I | | | | | | | |  |
| **3.21** | | Renal replacement therapy? | | Y | | | N | | Y | | | | | N | | Y | | | | | N | | |  |
| **3.22** | | Mechanical ventilation? (Include NIPPV but not mask CPAP) | | Y | | | N | | Y | | | | | N | | Y | | | | | N | | |  |
| **3.23** | | ICP monitor? | | Y | | | N | | Y | | | | | N | | Y | | | | | N | | |  |
| **3.24** | | ICP (mmHg) | | I__I__I | | | N/A | | I__I__I | | | | | N/A | | I__I__I | | | | | N/A | | |  |
| **3.25** | | Receiving vasoconstrictor or inotropic agent infusion? | | Y | | | N | | Y | | | | | N | | Y | | | | | N | | |  |
|  | | If answered **Yes** to question **3.25**, answer questions **3.26** to **3.35**; Otherwise go to question **3.36** | | | | | | | | | | | | | | | | | | | | | |  |
|  | | **3.26** | Noradrenaline | Y | | | N | | Y | | | | | N | | Y | | | | | N | | |  |
|  | | **3.27** | Adrenaline | Y | | | N | | Y | | | | | N | | Y | | | | | N | | |  |
|  | | **3.28** | Vasopressin | Y | | | N | | Y | | | | | N | | Y | | | | | N | | |  |
|  | | **3.29** | Dopamine | Y | | | N | | Y | | | | | N | | Y | | | | | N | | |  |
|  | | **3.30** | Dobutamine | Y | | | N | | Y | | | | | N | | Y | | | | | N | | |  |
|  | | **3.31** | Milrinone | Y | | | N | | Y | | | | | N | | Y | | | | | N | | |  |
|  | | **3.32** | Phenylephrine | Y | | | N | | Y | | | | | N | | Y | | | | | N | | |  |
|  | | **3.33** | Metaraminol | Y | | | N | | Y | | | | | N | | Y | | | | | N | | |  |
|  | | **3.34** | Other, specify____________ | Y | | | N | | Y | | | | | N | | Y | | | | | N | | |  |
|  | | **3.35** | Other, specify____________ | Y | | | N | | Y | | | | | N | | Y | | | | | N | | |  |
| ***RESUSCITATION EPISODE*** | | | | ***Episode 1*** | | | | | ***Episode 2*** | | | | | | | ***Episode 3*** | | | | | | | |  |
| **3.36** | | Heart rate (bpm) | | I__I__I__I | | | | | I__I__I__I | | | | | | | I__I__I__I | | | | | | | |  |
| **3.37** | | MAP (mmHg) | | I__I__I__I | | | | | I__I__I__I | | | | | | | I__I__I__I | | | | | | | |  |
| **3.38** | | Systolic ABP (mmHg) | | I__I__I__I | | | | | I__I__I__I | | | | | | | I__I__I__I | | | | | | | |  |
| **3.39** | | Diastolic ABP (mmHg) | | I__I__I__I | | | | | I__I__I__I | | | | | | | I__I__I__I | | | | | | | |  |
| **3.40** | | CVP (mmHg) | | I__I__I | | | | | I__I__I | | | | | | | I__I__I | | | | | | | |  |
| **3.41** | | PCWP (mmHg) | | I__I__I | | | | | I__I__I | | | | | | | I__I__I | | | | | | | |  |
| **3.42** | | Hb (g/L) (Only record Hb from ABG if transfused based on that Hb) | | I__I__I__I | | | | | I__I__I__I | | | | | | | I__I__I__I | | | | | | | |  |
| **3.43** | | Creatinine (μmol/L) | | I__I__I__I__I | | | | | I__I__I__I__I | | | | | | | I__I__I__I__I | | | | | | | |  |
| **3.44** | | Bilirubin (μmol/L) | | I__I__I__I | | | | | I__I__I__I | | | | | | | I__I__I__I | | | | | | | |  |
| **3.45** | | Platelet count (10^9^/L) | | I__I__I__I__I | | | | | I__I__I__I__I | | | | | | | I__I__I__I__I | | | | | | | |  |
| **3.46** | | Base excess | | I__I__I.I__I | | | | | I__I__I.I__I | | | | | | | I__I__I.I__I | | | | | | | |  |
| **3.47** | | Base deficit | | I__I__I.I__I | | | | | I__I__I.I__I | | | | | | | I__I__I.I__I | | | | | | | |  |
| **3.48** | | Lactate (mmol/L) | | I__I__I.I__I | | | | | I__I__I.I__I | | | | | | | I__I__I.I__I | | | | | | | |  |
| **3.49** | | Serum Albumin (g/L) | | I__I__I | | | | | I__I__I | | | | | | | I__I__I | | | | | | | |  |
| **3.50** | | INR | | I__I__I.I__I | | | | | I__I__I.I__I | | | | | | | I__I__I.I__I | | | | | | | |  |
| **3.51** | | APTT (seconds) | | I__I__I__I | | | | | I__I__I__I | | | | | | | I__I__I__I | | | | | | | |  |
| **Fluid output:** | | | | | | | | | | | | | | | | | | | | | | | |  |
| **3.52** | | Urine output (mL) previous complete hour | | I__I__I__I__I | | | | | I__I__I__I__I | | | | | | | I__I__I__I__I | | | | | | | |  |
| **3.53** | | Total fluid output (mL) previous complete hour | | I__I__I__I__I | | | | | I__I__I__I__I | | | | | | | I__I__I__I__I | | | | | | | |  |
| ***RESUSCITATION EPISODE*** | | | | | | | ***Episode 1*** | | | | | | ***Episode 2*** | | | | | | | ***Episode 3*** | | | |  |
| **Crystalloids received as boluses:** | | | | | | | | | | | | | | | | | | | | | | | |  |
| **3.54** | | Normal saline (mL) | | | | | I__I__I__I__I__I | | | | | | | I__I__I__I__I__I | | | | | | | I__I__I__I__I__I | | |  |
| **3.55** | | Hypertonic saline (mL) (>0.9% NaCl) | | | | | I__I__I__I__I__I | | | | | | | I__I__I__I__I__I | | | | | | | I__I__I__I__I__I | | |  |
| **3.56** | | 5% glucose or glucose saline mixture (eg 4% and N/5 saline) (mL) | | | | | I__I__I__I__I__I | | | | | | | I__I__I__I__I__I | | | | | | | I__I__I__I__I__I | | |  |
| **3.57** | | Hypertonic glucose (mL) (>5%) | | | | | I__I__I__I__I__I | | | | | | | I__I__I__I__I__I | | | | | | | I__I__I__I__I__I | | |  |
| **3.58** | | Balanced salt solution (mL) | | | | | I__I__I__I__I__I | | | | | | | I__I__I__I__I__I | | | | | | | I__I__I__I__I__I | | |  |
| **3.59** | | Other (mL), Specify:_____________________ | | | | | I__I__I__I__I__I | | | | | | | I__I__I__I__I__I | | | | | | | I__I__I__I__I__I | | |  |
| **Colloids received as boluses:** | | | | | | | | | | | | | | | | | | | | | | | |  |
| **3.60** | | Albumin 4-5% (mL) | | | | | I__I__I__I__I__I | | | | | | | I__I__I__I__I__I | | | | | | | I__I__I__I__I__I | | |  |
| **3.61** | | Albumin 20-25% (mL) | | | | | I__I__I__I__I__I | | | | | | | I__I__I__I__I__I | | | | | | | I__I__I__I__I__I | | |  |
| **3.62** | | Hetastarch (mL) | | | | | I__I__I__I__I__I | | | | | | | I__I__I__I__I__I | | | | | | | I__I__I__I__I__I | | |  |
| **3.63** | | Pentastarch (mL) | | | | | I__I__I__I__I__I | | | | | | | I__I__I__I__I__I | | | | | | | I__I__I__I__I__I | | |  |
| **3.64** | | Hextend (mL) | | | | | I__I__I__I__I__I | | | | | | | I__I__I__I__I__I | | | | | | | I__I__I__I__I__I | | |  |
| **3.65** | | Hespan (mL) | | | | | I__I__I__I__I__I | | | | | | | I__I__I__I__I__I | | | | | | | I__I__I__I__I__I | | |  |
| **3.66** | | Haesteril 6% (mL) | | | | | I__I__I__I__I__I | | | | | | | I__I__I__I__I__I | | | | | | | I__I__I__I__I__I | | |  |
| **3.67** | | Haesteril 10% (mL) | | | | | I__I__I__I__I__I | | | | | | | I__I__I__I__I__I | | | | | | | I__I__I__I__I__I | | |  |
| **3.68** | | Voluven | | | | | I__I__I__I__I__I | | | | | | | I__I__I__I__I__I | | | | | | | I__I__I__I__I__I | | |  |
| **3.69** | | 706 plasma replacement (mL) | | | | | I__I__I__I__I__I | | | | | | | I__I__I__I__I__I | | | | | | | I__I__I__I__I__I | | |  |
| **3.70** | | Hemohes/Starquin 6% (mL) | | | | | I__I__I__I__I__I | | | | | | | I__I__I__I__I__I | | | | | | | I__I__I__I__I__I | | |  |
| **3.71** | | Hemohes/Starquin 10% | | | | | I__I__I__I__I__I | | | | | | | I__I__I__I__I__I | | | | | | | I__I__I__I__I__I | | |  |
| **3.72** | | Other starch, specify___________________ | | | | | I__I__I__I__I__I | | | | | | | I__I__I__I__I__I | | | | | | | I__I__I__I__I__I | | |  |
| **3.73** | | Gelofusine (mL) | | | | | I__I__I__I__I__I | | | | | | | I__I__I__I__I__I | | | | | | | I__I__I__I__I__I | | |  |
| **3.74** | | Haemaccel (mL) | | | | | I__I__I__I__I__I | | | | | | | I__I__I__I__I__I | | | | | | | I__I__I__I__I__I | | |  |
| **3.75** | | Other gelatin, specify___________________ | | | | | I__I__I__I__I__I | | | | | | | I__I__I__I__I__I | | | | | | | I__I__I__I__I__I | | |  |
| **3.76** | | Dextran 40 | | | | | I__I__I__I__I__I | | | | | | | I__I__I__I__I__I | | | | | | | I__I__I__I__I__I | | |  |
| **3.77** | | Dextran 70 | | | | | I__I__I__I__I__I | | | | | | | I__I__I__I__I__I | | | | | | | I__I__I__I__I__I | | |  |
| **3.78** | | Other dextran, specify__________________ | | | | | I__I__I__I__I__I | | | | | | | I__I__I__I__I__I | | | | | | | I__I__I__I__I__I | | |  |
| **Blood products received:** | | | | | | | | | | | | | | | | | | | | | | | |  |
| **3.79** | | Packed Cells/Whole Blood (mL) | | | | | I__I__I__I__I__I | | | | | | | I__I__I__I__I__I | | | | | | | I__I__I__I__I__I | | |  |
| **3.80** | | Fresh Frozen Plasma (mL) | | | | | I__I__I__I__I | | | | | | | I__I__I__I__I | | | | | | | I__I__I__I__I | | |  |
| **3.81** | | Platelets (mL) | | | | | I__I__I__I__I | | | | | | | I__I__I__I__I | | | | | | | I__I__I__I__I | | |  |

| **SAFE TRIPS** | **DAY SUMMARY FORM 4** |
| --- | --- |

| ***STUDY DAY FLUID TOTALS*** | | |
| --- | --- | --- |
| **4.01** | What was the total volume of crystalloid received by infusion during the study day? (do not include the first hour of an infusion of >5mL/kg/hr, record that first hour on form 3 or 3A) (mLs) | I__I__I__I__I__I |
| **4.02** | What was the total volume of enteral nutrition received during the study day? (mLs) | I__I__I__I__I__I |
| **4.03** | What was the total volume of parenteral nutrition received during the study day? (mLs) | I__I__I__I__I__I |
| **4.04** | What was the total volume of fluid input for the study day (mLs) | I__I__I__I__I__I |
| **4.05** | What was the total urine output for the study day? (mLs) | I__I__I__I__I__I |
| **4.06** | What was the total volume of fluid output for the study day? (mLs) | I__I__I__I__I__I |
| **4.07** | What was the total frusemide dose received during the study day? (mg in 24 hours) | I__I__I__I__I |
| **4.08** | What was the total volume of 4-5% albumin received as a continuous infusion during the study day (do not include the first hour of the infusion, record first hour on form 3 or 3A) (mLs) | I__I__I__I__I__I |
| **4.09** | What was the total volume of 20-25% albumin received as a continuous infusion during the study day (do not include the first hour of the infusion, record first hour on form 3 or 3A) (mLs) | I__I__I__I__I__I |
| **4.10** | What was the total volume of any other colloid received as a continuous infusion during the study day (do not include the first hour of the infusion, record first hour on form 3 or 3A) (mLs)  Write colloid type here: | I__I__I__I__I__I |
| **4.10** | What was the total volume of any other colloid received as a continuous infusion during the study day (do not include the first hour of the infusion, record first hour on form 3 or 3A) (mLs)  Write colloid type here: | I__I__I__I__I__I |
| **4.10** | What was the total volume of any other colloid received as a continuous infusion during the study day (do not include the first hour of the infusion, record first hour on form 3 or 3A) (mLs)  Write colloid type here: | I__I__I__I__I__I |

| **SAFE TRIPS** | **OUTCOME SUMMARY FORM 5** |
| --- | --- |

| *DISCHARGE / DEATH* | | | | | | |
| --- | --- | --- | --- | --- | --- | --- |
|  | **yes** | | **no** |  | | |
| **5.01** | Y | | N | By the end of day 28 (since ICU admission), has the patient been discharged (alive or deceased) from ICU? **If yes, GO TO 5.02; If no, form 5 is complete** | | |
| **5.02** | I__I__I/I__I__I/I__I__I__I__I | | | | | What was the date of ICU discharge? **GO TO 5.03;** |
| **5.03** | Y | | N | Was the patient alive at ICU discharge? If **yes**, **GO TO 5.04; If no, form 5 is complete** | | |
| **5.04** | Y | | N | By the end of day 28 (since ICU admission), has the patient been discharged (alive or deceased) from hospital? **If yes, GO TO 5.05; If no, form 5 is complete** | | |
| **5.05** | I__I__I/I__I__I/I__I__I__I__I | | | | | What was the date of hospital discharge? **GO TO 5.06;** |
| **5.06** | Y | N | | | Was the patient alive at hospital discharge? | |

| **SAFE TRIPS** | **Bed-side data collection form** |
| --- | --- |

SAFE TRIPS is a multi-national study collecting data on fluid resuscitation practices in the ICU and will be conducted on a single 24 hour period beginning on either May 16 or June 20, 2007. At our hospital, this will be from ______________________________ to _____________________________________

We would like to thank you for your involvement.

Please assist us by recording information on types of **boluses** of fluid including **blood products** given to expand or maintain the intravascular volume. Please note we **do not** wish you to record types of fluid given as maintenance or drug infusions. A resuscitation episode is any individual hour when a bolus of crystalloid is given, or any colloid or blood product is given, or the first hour of a colloid infusion or the first hour of a crystalloid infusion of 5mL/kg/hour or greater.

For each episode please record the

1. reason (indication the fluid was given),
2. classification of the staff member who ordered or prescribed the fluid.
3. Type of fluid that was given (crystalloid, colloid or blood product)

If you are uncertain about these details please clarify with the person who ordered or prescribed the fluid or with ­_______________________________.

| **Crystalloid solutions include the following** | |
| --- | --- |
| - Normal saline | - Hypertonic glucose (>5%) |
| - Hypertonic saline (>0.9% NaCl) | - Balanced salt solution (Hartmanns, Ringers etc.) |
| - 5% glucose or glucose saline mixture (e.g. 4% and N/5 saline) |  |

| **Colloid solutions include the following** | |
| --- | --- |
| - Albumin 4-5% or Albumin 20-25% | - 706 plasma replacement |
| - Hetastarch | - Hemohes/Starquin 6% or Hemohes/Starquin 10% |
| - Pentastarch |  |
| - Hextend | - Gelosfusine |
| - Hespan | - Haemaccel |
| - Haesteril 6% or Haesteril 10% | - Dextran 40 or Dextran 70 |
| - Voluven |  |

| **Blood products include the following** |
| --- |
| - Packed Cells/Whole Blood |
| - Fresh Frozen Plasma |
| - Platelets |

**Please do not file this information in the patient’s history. The forms will be collected by the investigators at your hospital**

| ***RESUSCITATION EPISODE*** | | | ***Episode ___*** | ***Episode ___*** | | ***Episode ___*** | |
| --- | --- | --- | --- | --- | --- | --- | --- |
| Q1 Start time of fluid bolus (24 hr clock) | | I__I__I:I__I__I | | I__I__I:I__I__I | | I__I__I:I__I__I | |
| **Q2 What were the indications for fluid for this resuscitation episode? (more than one box may be ticked)** | | | | | | | |
| 2.1 | Hypotension | | 🞏 | | 🞏 | | 🞏 |
| 2.2 | Increasing or persisting inotrope or vasopressor requirements | | 🞏 | | 🞏 | | 🞏 |
| 2.3 | Low CVP | | 🞏 | | 🞏 | | 🞏 |
| 2.4 | Low PCWP (wedge pressure) or PAOP | | 🞏 | | 🞏 | | 🞏 |
| 2.5 | Tachycardia | | 🞏 | | 🞏 | | 🞏 |
| 2.6 | Low urine output | | 🞏 | | 🞏 | | 🞏 |
| 2.7 | Low measured cardiac output | | 🞏 | | 🞏 | | 🞏 |
| 2.8 | Low Hb concentration | | 🞏 | | 🞏 | | 🞏 |
| 2.9 | Low S_v_O_2_/S_cv_O_2_ | | 🞏 | | 🞏 | | 🞏 |
| 2.10 | Ongoing bleeding | | 🞏 | | 🞏 | | 🞏 |
| 2.11 | Other ongoing fluid loss | | 🞏 | | 🞏 | | 🞏 |
| 2.12 | Unit protocol or standing orders | | 🞏 | | 🞏 | | 🞏 |
| 2.13 | Evidence of poor peripheral perfusion | | 🞏 | | 🞏 | | 🞏 |
| 2.14 | Increasing or persisting acidosis or lactate | | 🞏 | | 🞏 | | 🞏 |
| 2.15 | Other, Specify _____________________ | | 🞏 | | 🞏 | | 🞏 |
| **Q3 Who decided the choice of fluid for this resuscitation episode? (tick one box only per resuscitation episode)** | | | | | | | |
| 3.1 | ICU doctor | | 🞏 | 🞏 | | 🞏 | |
| 3.2 | Surgical doctor | | 🞏 | 🞏 | | 🞏 | |
| 3.3 | Medical doctor | | 🞏 | 🞏 | | 🞏 | |
| 3.4 | Nurse acting independently | | 🞏 | 🞏 | | 🞏 | |
| 3.5 | Nurse following unit protocol | | 🞏 | 🞏 | | 🞏 | |
| 3.6 | Other | | 🞏 | 🞏 | | 🞏 | |
| **Q4 If you chose ICU, surgical or medical doctor on Q. 3, specify the doctor’s level (tick one box only per resuscitation episode); Otherwise, go to question Q. 5** | | | | | | | |
| 4.1 | Specialist/consultant/attending | | 🞏 | 🞏 | | 🞏 | |
| 4.2 | Registrar/Fellow/Senior Trainee | | 🞏 | 🞏 | | 🞏 | |
| 4.3 | Resident/HMO/Junior Trainee | | 🞏 | 🞏 | | 🞏 | |
| 4.4 | Intern/House officer | | 🞏 | 🞏 | | 🞏 | |
| **Q5 Please indicate the type of fluid that was given. Please tick all options that apply** | | | | | | | |
| 5.1 | Crystalloid solution | | 🞏 | 🞏 | | 🞏 | |
| 5.2 | Colloid solution | | 🞏 | 🞏 | | 🞏 | |
| 5.3 | Blood product | | 🞏 | 🞏 | | 🞏 | |
