# Supplementary material for: Resuscitation fluid use in critically ill adults: an international cross-sectional study in 391 intensive care units
Source: Crit Care. 2010 Oct 15;14(5):R185. doi: 10.1186/cc9293 (PMC3219291; doi:10.1186/cc9293)
Supplement: Additional file 2 — Hierarchy for indications: Hierarchy of indications for administration of resuscitation fluid. Were more than one indication was given the indication highest on the hierarchy was taken to be the main indication. [file cc9293-S2.DOCX]

**Additional File 2**: Hierarchy for indications

1. Impaired perfusion OR low cardiac output

YES to any of: Low urine output (3.07)

Low measured cardiac output (3.08)

Low SvO2/ScvO2 (3.10)

Evidence of poor peripheral perfusion (3.14)

Increasing or persisting acidosis or lactate (3.15)

1. Anaemia OR bleeding OR coagulopathy OR thrombocytopaenia

NO to all of: Low urine output (3.07)

Low measured cardiac output (3.08)

Low ScO2/ScvO2 (3.10)

Evidence of poor peripheral perfusion (3.14)

Increasing or persisting acidosis or lactate (3.15)

YES to any of: Low Hb concentration (3.09)

Ongoing bleeding (3.11)

‘Coagulopathy’ (3.16)

‘Thrombocytopaenia’ (3.16)

1. Other fluid losses

NO to all of: Low urine output (3.07)

Low measured cardiac output (3.08)

Low ScO2/ScvO2 (3.10)

Evidence of poor peripheral perfusion (3.14)

Increasing or persisting acidosis or lactate (3.15)

Low Hb concentration (3.09)

Ongoing bleeding (3.11)

‘Coagulopathy’ (3.16)

‘Thrombocytopaenia’ (3.16)

YES to any of: Other ongoing fluid loss (3.12)

1. Unit protocol

NO to all of: Low urine output (3.07)

Low measured cardiac output (3.08)

Low ScO2/ScvO2 (3.10)

Evidence of poor peripheral perfusion (3.14)

Increasing or persisting acidosis or lactate (3.15)

Low Hb concentration (3.09)

Ongoing bleeding (3.11)

‘Coagulopathy’ (3.16)

‘Thrombocytopaenia’ (3.16)

Other ongoing fluid loss (3.12)

YES to any of: Unit protocol or standing orders (3.13)

1. Abnormal vital signs only

NO to all of: Low urine output (3.07)

Low measured cardiac output (3.08)

Low Hb concentration (3.09)

Low ScO2/ScvO2 (3.10)

Ongoing bleeding (3.11)

Other ongoing fluid loss (3.12)

Unit protocol or standing orders (3.13)

Evidence of poor peripheral perfusion (3.14)

Increasing or persisting acidosis or lactate (3.15)

Other (3.16)

YES to any of: Hypotension (3.02)

Increasing inotrope or vasopressor requirements (3.03)

Low CVP (3.04)

Low PCWP (3.05)

Tachycardia (3.06)

1. Other

NO to: 3.02 – 3.15

YES to: 3.16 other than ‘coagulopathy’ or ‘thrombocytopaenia’
