# Supplementary material for: Resuscitation fluid use in critically ill adults: an international cross-sectional study in 391 intensive care units
Source: Crit Care. 2010 Oct 15;14(5):R185. doi: 10.1186/cc9293 (PMC3219291; doi:10.1186/cc9293)
Supplement: Additional file 3 — The SAFE TRIPS Investigators: Listing of all SAFE TRIPS investigators by country and institution. [file cc9293-S3.DOCX]

**Additional File 3:** The SAFE TRIPS Investigators

***Writing committee*:** S. Finfer (Chair), B. Liu, C. Taylor, R. Bellomo, L. Billot, D. Cook, B Du, C. McArthur, J. Myburgh, ***Management committee*:** S. Finfer (chair), Y. An, L. Billot, R. Bellomo, J. Cooper, B. Du, M. Fitzharris, D. Gantner, A. Higgins, B. Liu, C. McArthur, S. McEvoy, J. Myburgh, C. Taylor. ***Investigators and participating centres*:** ***Australia (Country Coordinator: S Finfer):*** *Alice Springs Hospital –* S Jacobs; *Ballarat Base Hospital –* R. Gazzard; *Bendigo Hospital –* J Edington; *Blacktown Hospital –* D Ghelani; *Freemantle Hospital –* D Blythe; *Gold Coast Hospital –* B Richards; *Lismore Hospital –* C McCalman; *Liverpool Hospital –* M Parr; *Monash Medical Centre –* C Walker; *Nepean Hospital –* I Seppelt, L Cole; *Royal Darwin Hospital –* D Stevens; *Royal Melbourne Hospital –* J Cade; *Royal North Shore Hospital –* S Finfer; *Royal Perth Hospital –* S Webb; *Royal Prince Alfred –* C Woolfe; *Sir Charles Gairdner Hospital –* PV van Heerden; *St George Hospital –* J Myburgh; *The Alfred Hospital –* J Cooper; *The Austin Hospital –* R Bellomo; *The Canberra Hospital –* I Mitchell; *The Queen Elizabeth Hospital –* S Peake; *The Western –* C French; *Westmead Hospital Cardiovascular –* H Playford; *Westmead Hospital Other –* A Bannerjee. ***Brazil (Country Coordinator: O Berwanger):*** *Hospital Moinhos de Vento –* N da Silva; *Hospital Português –* J Mario Teles; *Hospital São Paulo –* H Guimarães; *Pavilhão Pereira Filho –* M Rocha; *Vitória Apart Hospital –* C Piras. ***Canada (Country Coordinator: L McIntyre & D Cook):*** *Alberta University Hospital –* SM Bagshaw; *Calgary Health Region Calgary –* T Stelfox; *Capital Health - QE II Health Sciences Centre –* R Green & R Hall; *Chilliwick ICU –* W Son; *Hamilton General –* D Cook, M Meade; *Health Sciences Center Winnipeg –* A Kumar; *Henderson Hospital Hamilton –* D Cook, M Meade; *Langley ICU –* R Wittman; *London Health Sciences Centre University Hospital –* C Martin; *London Health Sciences Centre Victoria Hospital –* C Martin; *Maisonneuve Rosemont Montreal –* M Leblanc; *McMaster Hospital Hamilton –* D Cook, M Meade; *MSA ICU –* S Lim; *Royal Columbian Hospital –* S Keenan; *Royal Vic Montreal –* S Magder; *St Boniface Hospital Winnipeg –* A Kumar; *St Joseph Hospital Hamilton –* D Cook, M Meade; *The Ottawa Hospital Civic Campus –* L McIntyre; *The Ottawa Hospital General Campus –* L McIntyre; *Vancouver General Hospital –* D Chittock. ***China (Country Coordinator: B Du & Y An):*** *Beijing Cancer Hospital –* H Wang, J Dong; *Beijing Chaoyang Hospital of Capital University of Medical Sciences –* Wenxiong Li, X Chen; *Beijing Friendship Hospital of Capital University of Medical Sciences –* A Li, H Zhuang; *Beijing General Hospital of Beijing Military Command –* W Liu; *Beijing Hospital –* Y Liu, Q He, J Wang; *Beijing Ji Shui Tan Hospital –* N Zhou, Y Bai; *Beijing PLA 304 Hospital –* L Wang; *Beijing Shijitan Hospital –* F Lu, W Chen, S Wang; *Beijing Tiantan Hospital of Capital University of Medical Sciences –* J Zhou, Z Zhang; *Beijing Tongren Hospital of Capital University of Medical Sciences –* Y Xu, T Li; *Bengbu Municipal Central Hospital –* X Sun, B Wang; *China Medical University First Hospital –* Z Zhang, C Zhao; *China PLA 301 General Hospital –* Q Song, L Pan; *China PLA 309 Hospital –* P Ma, Q Li; *China-Japan Friendship Hospital –* G Li, D Chen; *Dalian Municipal Central Hospital –* L Liu, K Gao, S Han; *First affiliated Hospital of Dalian Medical University –* X Wan, Y Zhang; *First Hospital of Tsinghua University –* Y Chao, L Wang; *Fuxing Hospital of Capital University of Medical Sciences –* X Xi, L Jiang, C Han; *Guangdong General Hospital –* T Qin, S Wang, W Jiang; *Guangzhou Institute of Respiratory Diseases, The First Affiliated Hospital of Guangzhou Medical University –* Y Li, X Liu; *Hebei Medical University Forth Hospital –* Z Hu, Y Ding; *Nanjing Jinling Hospital, Nanjing University –* W Li, X Wang; *Navy General Hospital –* Z Zhang, Y Liu; *Peking Union Medical College Hospital –* B Du, L Weng; *Peking University First Hospital –* D Wang, S Li; *Peking University People's Hospital –* Y An, Z Zhang; *Peking University Shenzhen Hospital –* W Zhang, H Luo; *Peking University Shougang Hospital –* Y Luo; *Peking University Third Hospital –* X Zhu, H Li; *Qilu Hospital of Shandong University –* C Li, Y Li; *Ruijin Hospital RICU) –* H Qu, W Chen; *Ruijin Hospital (SICU) –* E Mao, D Min; *Shandong Provincial Hospital –* C Wang, J Zhang, H Ren; *Shanxin Medical University First Hospital –* H Liu, M Wang; *The First Affiliated Hospital of Harbin Medical University –* M Zhao, D Fei; *The First affiliated Hospital of Kunming Medical College –* C Qian, R Liu; *The First Hospital of Lanzhou University –* B Shi, H Guo; *The First Hospital, Sun Yat-Sun University –* X Guan, J Wu; *The First People's Hospital of Foshan, Giangdong –* L Zhou, Y Li; *The Second Affiliated Hospital of Harbin Medical University –* K Yu, H Wang; *The Second affiliated Hospital of Jilin University –* Y Wang, D Li; *The Second affiliated Hospital of Kunming Medical College –* Q Huang, M Su; *The Second Hospital of Lanzhou University –* C Dong; *The Tenth People's Hospital of Shanghai –* X Zhang; *The Third Hospial of Xiamen –* B Wu; *Tianjin Third Center Hospital –* Y Qin, N Zhang; *West China Hospital of Sichuan University –* Y Kang, B Wang, Y Deng; *Xiangya hospital of centre-south university –* Y Ai, Y Guo; *Xijing Hospital of the Fourth Military Medical University (CICU) –* Q Cui *Xijing Hospital of the Fourth Military Medical University (SICU) –* X Zhang; *Xuanwu Hospital of Capital University of Medical Sciences –* J Jia, H Chen; *Zhejiang Hospital –* J Yan, Q Xu; *Zhejiang Provincial People's Hospital –* R Sun, J Hong; *Zhejiang University First Hospital –* Q Fang, X Zheng; *Zhongda Hospital, Southeast University –* H Qiu, S Liu; *Zhongnan Hospital of Wuhan University –* Qi Zhou, J Li. ***Denmark (Country Coordinator – A Perner):*** *Aarhus Sygehus TGH –* N Schonemann; *Amager Hospital –* A Bendtsen; *Gentofte Hospital –* K Thornberg; *Glostrup Hospital –* H Boensen; *Herlev Hospital –* H Tousi; *Hillerod Hospital –* M Bestle; *Hjorring Sygehus –* M Pawlowicz; *Holbaek Sygehus –* D Høen-Beck; *Hvidovre Hospital –* P Carl; *Kolding Sygehus –* E Ronholm; *Neurocentret Rigshospitalet –* K Welling; *Regionshospitalet Herning –* J Strelitz; *Regionshospitalet Holstebro –* C Kancir; *Regionshospitalet Horsens –* A Hostrup; *Rigshospitalet –* A Perner; *Skejby Sygehus –* R Jensen; *Svendborg Sygehus –* J Westergard-Nielsen. ***England / Wales (Country Coordinator – J D Young & B Cutherbertson):*** *Castle Hill –* S Bennett; *Dorset County Hospital –* A Ball; *Dudely Group of Hospitals, Russells Hall Hospital Dudley –* H Becker; *East Surrey Hospital Redhill –* S Desikan; *Eastbourne District General Hospital –* N Watson; *Homerton Hospital –* D Watson; *Hull Royal Infirmary –* I Smith; *James Paget University Hospitals –* M Wright; *John Radcliffe Hospital –* J Millo; *Kent and Canterbury Hospital –* J Morris; *Macclesfield District General Hospital –* A Williams; *Mayday Hospital –* A Peebles-Brown; *Musgrove Park Hospital –* K Grainger; *Northampten General Hospial –* R Marsh; *Princess Royal Hospital –* D Christmas; *Rotherham District General –* D Harling; *Royal Devon & Exeter NHS Foundation Trust –* C Boulanger; *Royal Free Hospital –* A Davenport; *Royal Hants County Hospital –* A Goldsmith; *Royal Infirmary of Edinburgh –* B Cook; *Royal Sussex County Hospital –* S Drage; *Salford Royal Hospital –* J Goodall; *Southend University Hospital –* D Higgins; *Southmead Hospital –* J Price; *St Richard's Hospital –* M Margarson; *St Thomas Hospital –* T Sherry; *The Queen Elizabeth Hospital –* F McAuley; *Wansbeck General Hospital –* A Syndercombe; *Withybush General –* G Jones; *Yeovil District Hospital –* J Reid. ***France (Country Coordinator – F Schortgen):*** *CH Bligny –* P Andrivet; *CH de Dourdan –* S Jamali; *CH Dieppe –* J Rigaud; *CH Etampes –* A Gaffine; *CH Lhomme –* N Kerkeni; *CH Macon –* C Mejean; *CH Pau –* J Drault; *CH Roanne –* P Beuret; *CHI Elbeuf-Louviers –* B Bourffandeau; *CHI Font Pré-Toulon –* J Gasselin; *CHI Poissy-Saint Germain –* B de Jonghe; *CHU d'Angers –* A Mercat; *CHU de Dijon –* J Quenot; *CHU de Grenoble –* C Broux; *CHU de Grenoble –* J Timsit; *Hôpital André Mignot –* H Mokhtar; *Hôpital Antoine Beclère-APHP –* F Jacobs; *Hôpital Beaujon-PHP –* S Pease; *Hôpital Bichat-APHP –* B Mourvillier; *Hôpital Bichat-APHP –* S Lasocki; *Hôpital Charles Nicole CHU de Rouen –* K Clabault; *Hôpital Civil CHU de Strasbourg –* H Rahmani; *Hôpital Cochin-Port Royal-APHP –* A Cariou; *Hôpital de la Croix Rousse CHU Lyon –* C Guerin; *Hôpital de la Pitié Salpetrière-APHP –* A Combes; *Hôpital de la Pitié Salpetrière-APHP –* Al Duguet; *Hôpital de Lafontaine-Saint Denis –* M Thuong; *Hôpital Haut Lévêque CHU de Bordeaux –* G Janvier; *Hôpital Henri Mondor-APHP –* F Schortgen; *Hôpital L'archet CHU Nice –* C Icahai; *Hôpital Lariboisière-APHP –* B Megarbane; *Hôpital Lariboisière-APHP –* D Payen; *Hôpital Louis Mourrier-APHP –* R Leon; *Hôpital Pellegrin Tripode CHU de Bordeaux –* D Gruson; *Hôpital saint Antoine-APHP –* B Guidet;*Hôpital Saint Camille-Bry sur Marne –* D Tardu; *Hôpital Sainte- Marguerite CHU Marseille –* A Roch; *Hôpital Tenon-APHP –* C Ridel; *Hôpital Tenon-APHP –* M Fartoukh; *Hôpital Victor Dupouy-Argenteuil –* H Mentec; *Hôtel Dieu CHU de Nantes –* C Guitton; *Instutut Gustave Roussy-Villejuif –* F Blot. ***Germany (Country Coordinator – F M Brunkhorst):*** *Charité - Universitätsmedizin Berlin Campus Virchow-Klinikum Medizinische Klinik mit Schwerpunkt Nephrologie –* M Oppert; *Charité - Universitätsmedizin Berlin Universitätsklinik für Anästhesiologie und operative Intensivmedizin Charité Campus Mitte Klinikum –* C Spies; *Charité - Universitätsmedizin Berlin Universitätsklinik für Anästhesiologie und operative Intensivmedizin Charité Campus Virchow Klinikum –* C Spies; *Ernst-Moritz-Arndt-Universität Greifswald, Klinik und Poliklinik für Anästhesie und Intensivmedizin –* M Gründling; *Ernst-Moritz-Arndt-Universität Greifswald, Zentrum für Innere Medizin B –* S Friesecke; *HELIOS Klinikum Erfurt, Klinik für Anästhesiologie und Intensivtherapie –* A Meier-Hellmann; *Klinik am Eichert, Klinik für Anästhesiologie, operative Intensivmedizin und Schmerztherapie –* J Martin; *Klinikum Augsburg, Klinik für Anästhesiologie und Intensivtherapie –* U Jaschinski; *Klinikum der Universität München, Medizinische Klinik - Innenstadt, Intensivmedizin –* R Gärtner; *Klinikum Oldenburg Klinik für Anästhesiologie, Intensivmedizin, Notfallmedizin, Schmerztherapie –* W Weyland; *Krankenhaus Köln-Merheim, Klinik für Anästhesiologie und operative Intensivmedizin –* F Wappler; *Martin-Luther-Universität Halle-Wittenberg Universitätsklinik für Anästhesiologie und operative Intensivmedizin –* H Bromber; *Medizinische Hochschule Hannover, Zentrum für Innere Medizin/ Pneumologie –* T Welte, J Hadem; *St. Elisabeth Krankenhaus Köln-Hohenlind, Klinik für Anästhesiologie und operative Intensivmedizin –* F Fiedler; *Städtisches Klinikum München GmbH - Klinikum Harlaching Klinik für Internistische Akutmedizin und Prävention –* C Peckelsen; *Städtisches Krankenhaus Martha-Maria Halle-Dölau gGmbH Klinik für Anästhesiologie und Intensivmedizin –*H Fritz; *Universitätsklinikum des Saarlandes Klinik für Anästhesiologie, Intensivmedizin und Schmerztherapie –* H Rensing; *Universitätsklinikum Dresden, Klinik für Anästhesiologie und Intensivtherapie –* M Ragaller; *Universitätsklinikum Jena, Klinik für Anästhesiologie und Intensivtherapie –* K Reinhart, F M Brunkhorst; *Universitätsklinikum Leipzig, Klinik für Anästhesiologie und Intensivtherapie –* Albert; *Universitätsklinikum Tübingen, Medizinische Klinik, Otfried-Müller-Str. 10 und Klinik für Anästhesiologie und Intensivmedizin –* R Riessen; *Vivantes Klinikum Neukölln, Klinik für Anästhesie, operative Intensivmedizin und Schmerztherapie –* H Gerlach; *First Medical Department, Medical Faculty Mannheim, University of Heidelberg –* U Hoffmann . ***Hong Kong (Country Coordinator – G Joynt):*** *Caritas Medical Centre (CMC) –* FL Chow; *North District Hospital (NDH) –* C Cheng; *Prince of Wales hospital (PWH) –* G Joynt; *Princess Margaret Hospital (PMH) & Yan Chai Hospital (YCH) –* T Buckley; *Queen Elizabeth Hospital (QEH) –* K W Auyeung; *Queen Mary Hospital (QMH) –* K Young; *Tseung Kwan O Hospital (TKO) –* C K Ching. ***Iceland (Country Coordinator – S Karason):*** *FSA University Hospital - Regional hospital Akureyri –* S Sigurdsson; *Landspitali University Hospital Fossvogur –* K Sigvaldason; *Landspitali University Hospital Hringbraut –* K Hreinsson. ***India (Country Coordinator – F Kapadia):*** *P D Hinduja National Hospital MUMBAI INDIA –* F Kapadia. ***Ireland and Northern Ireland (Country Coordinator – B Marsh & D McAuley on behalf of the Irish Critical Care Trials Group):*** *AMNCH Hospital –* M Donnelly; *Antrim Area Hospital –* R Bailie; *Cork University Hospital –* D Breen; *Galway University Hospital –* J Bates; *Mater Misericicordiae University Hospital Dublin –* B Marsh; *Midwestern Regional Hospital, Limerick –* C Motherway; *Royal Victoria Hospital, Belfast –* D McAuley; *Ulster Hospital –* J Trinder. ***Italy (Country Coordinator – G Bertolini & C Smeraldi):*** *A. Manzoni –* G Mottura; *A.O. Carlo Poma Mantova - P.O. Destra Secchia –* O Andrei; *A.O. di Padova –* G Bonaccorso; *A.O. di Verona, Ospedale Maggiore –* A Luzzani; *A.O. G. Rummo –* E De Blasio; *Anna Rizzoli - Ischia –* R Buonanno; *AORN Cardarelli –* M De Cristofaro; *AORN Cardarelli –* M De Cristofaro; *AORNAS Garibaldi, S.Luigi-Currò, Ascoli-Tomaselli –* Pasqualino; *ARNAS Civico e Benfratelli –* E Mazzola; *Azienda Ospedaliera Universitaria Senese Policlinico Santa Maria alle Scotte –* M Monfregola; *Azienda Ospedaliera Villa Scassi –* M Isetta; *Azienda Universitaria Ospedaliera Senese –* F Franchi; *Cardinale G. Panìco –* F Trisolino; *Città di Sesto San Giovanni –* G Marchetti; *Civile –* G Piga; *Civile –* L Todesco; *Civile - ASL 19 –* S Perno; *Civile - ULSS 8 –* A Bianchin; *Civile "SS. Filippo e Nicola" –* A Blasetti; *Civile G. Salvini –* S Rossi; *Civile Regione Piemonte - ASL 9 –* R Salcuni; *Civile SS. Annunziata –* A Meloni; *di Ariano Irpino –* M Greco; *di Desio –* E Beck; *di Manerbio –* B Antonini; *di S. Chiara –* P Malacarne; *Fondazione Ospedale Maggiore Policlinico Mangiagalli Regina Elena –* E Prandi; *Francesco Ferrari –* G Negro; *G. Brotzu –* G Cubeddu; *G. Pasquinucci –* E Ferrari; *IRCCS Policlinico Maggiore –* S Rotelli; *IRCCS Policlinico Maggiore –* M Savioli; *IRCCS Policlinico San Matteo –* T Mediani; *Istituto Nazionale Tumori –* E Tognoli; *Istituto Ospitaliero –* A Ribola; *Madonna delle Grazie –* L Laperchia; *Maggiore –* S Meinardi; *Maggiore C.A. Pizzardi –* F Cancellieri; *Marino - ASL 8 –* S Mancosu; *Mauriziano Umberto I –* V Segala; *Maurizio Bufalini –* E Gamberini; *P.O. San Bambino - A.O. Universitaria Vittorio Emanuele –* G Garofalo; *Policlinico Monteluce –* N Dentini; *Policlinico San Matteo IRCCS –* L Carnevale; *Policlinico Umberto I - Università degli Studi di Roma –* F Bilotta; *Regionale della Valle d'Aosta –* F Brunod; *Regionale S. Maria dei Battuti Cà Foncello –* L Casagrande; *Riuniti –* I Riva; *S. Anna –* D Osti; *S. Maria del Prato –* V Sitta; *San Biagio Domodossola ASL 14 Omegna –* S Alleva; *San Donato –* G Becattini; *San Giacomo - ASL 8 –* S Munaron; *San Giovanni Battista - Le Molinette –* R Cavallo; *San Giovanni Battista - Le Molinette –* A Marzullo; *San Giovanni Boscov –* F Ferrari; *San Giovanni di Dio e Ruggi D'Aragona –* G Calicchio; *San Leonardo –* M Sucre; *San Salvatore –* L Quattrocchi; *San Salvatore –* C Breschi; *Sant' Andrea - ASL 11 –* A Gratarola; *Santa Croce –* P Sciacca; *Santa Maria di Loreto –* M Postiglione; *Santa Maria Nuova - ASL 10 –* M Barattini; *Sant'Anna di Como - Presidio Ospedaliero di Menaggio –* M Rossi; *Sant'Antonio Abate –* C Falcelli; *SS. Annunziata –* M Coaloa; *ULSS 17 Veneto Stabilimento ospedaliero –* S Cattin; *Umberto I –* S Palmese; *Universitario Policlinico G. Martino –* A David; *Vittorio Emanuele –* P Calabrese. ***Japan (Country Coordinator – H Morimatsu):*** *Ehime University Hospital –* K Dote; *Okayama Medical Center –* I Ohashi; *Okayama University Hospital –* H Morimatsu; *Osaka University Hospital –* Y Goto; *Tsuyama Central Hospital –* S Hagioka. ***New Zealand (Country Coordinator – C McArthur):*** *Auckland City CVICU –* S McGuiness; *Auckland City DCCM –* C McArthur; *Christchurch –* A Gibson, S Henderson; *Hawkes Bay –* R Freebairn; *Middlemore –* T Williams; *Nelson –* Br King; *North Shore –* J Liang; *Waikato Hospital –* F Van Haren; *Wellington –* D Dinsdale. ***Norway (Country Coordinator – AB Guttormsen & E Helst):*** Due to local ethics requirements, site names are not disclosed. ***Portugal (Country Coordinator – I Serra):*** *Hospital Fernando Fonseca –* I Serra.

***Saudi Arabia (Country Coordinator – Y Arabi):*** *King Abdulaziz Medical City –* Y Arabi; *King Faisal Specialist Hospital & Research Center- Jeddah –* I Qushmaq; *King Faisal Specialist Hospital & Research Center- Riyadh –* N Abouchala, M Kherallah; *Riyadh Armed Forces Hospital –* Y Mandourah. ***Scotland (Country Coordinator – B Cuthbertson):*** *Aberdeen –* B Cuthbertson; *Dumfries –* P Willis; *Dundee –* S Cole; *Dunfermline –* M MacDougall; *Edinburgh Western General –* P Andrews; *Paisley –* D Alcorn; *Stirling –* C Carins; *Western Infirmary –* B Digby. ***Singapore (Country Coordinator –S Loo):*** *National University Hospital, Singapore –* CK Tan; *Singapore General Hospital –* P Lee; *Tan Tock Seng Hospital Surgical ICU –* Y Chan. ***Sweden (Country Coordinator – A Aneman):*** *Boras Hospital –* P Petersen; *Danderyd Hospital –* J Albert; *Falun Hospital –* P Guldbrand; *Halmstad Hospital –* M Juhlin-Dannfeldt; *Helsingborg Hospital –* N Nielsen; *Huddinge University Hospital –* H Hjelmqvist; *Jonkoping Hospital –* P Nordlund; *Kalmar Hospital –* J Berkius; *Karolinska University Hospital –* A Oldner, D Konrad; *Kungalv –* R Zatterman; *Linkoping University Hosptial –* K Metcalf; *Lund University Hospital –* H Friberg; *Malmo Hospital –* M Chew; *Pitea Alvdals Hospital –* KLindgren; *Sahlgrenska University Hospital –* A Aneman; *Skovde Hospital –* R Gatz; *Soderjukhuset Stockholm – N/A; St Goran Hospital –* H Blomqvist; *Sunderby Hospital –* I Wizelius; *Uddevalla Hospital –* M Andersson; *Umea University Hospital –* M Rodling-Wahlstrom; *Uppsala University Hospital –* H Stiernstrom; *Vaxjo Hospital –* P Lindgren; *Ystad Hospital –* T Elvstad, U Hyddmark. ***Switzerland (Country Coordinator – T Merz):*** *Bern, University Hospital, Department of Intensive Care Medicine –* T Merz; *Biel, Cantonal Hospital, Intensive Care Unit –* M Laube; *Luzern, Cantonal Hospital, Intensive Care Unit –* C Haberthuer; *Winterthur, Cantonal Hospital, Intensive Care Unit –* M Jaeggi; *Zurich, University Hospital, Intensive Care Unit Department of Internal Medicine –* M Maggiorini; *Zurich, University Hospital, Intensive Care Unit Department of Surgery –* J Stover. ***UAE (Country Coordinator – R Ahmed):*** *Rashid Hospital Trauma Center, Dubai-UAE –* R Ahmed. ***USA (Country Coordinator – N/A):*** *CCM UPMC –* J Kellum,R Murugan, A Salmon; *Mayo Clinic, Rochester –* N Vlahakis; *University of Texas Health Care Center, San Antonio –* S Cohn; *US Army –* K Chung.
